# Polymer modeling of the *E. coli* genome reveals the involvement of locus positioning and macrodomain structuring for the control of chromosome conformation and segregation

**DOI:** 10.1093/nar/gkt1005

**Published:** 2013-11-03

**Authors:** Ivan Junier, Frédéric Boccard, Olivier Espéli

**Affiliations:** ^1^Centre for Genomic Regulation (CRG), Dr. Aiguader 88, 08003 Barcelona, Spain, ^2^Universitat Pompeu Fabra (UPF), 08003 Barcelona, Spain, ^3^CGM-CNRS, Université Paris-Sud, 1 Avenue de la Terrasse, 91198 Gif sur Yvette, France and ^4^CIRB – Collège de France, 11 Place Marcelin Berthelot, 75231 Paris Cedex 05, France

## Abstract

The mechanisms that control chromosome conformation and segregation in bacteria have not yet been elucidated. In *Escherichia coli*, the mere presence of an active process remains an open question. Here, we investigate the conformation and segregation pattern of the *E. coli* genome by performing numerical simulations on a polymer model of the chromosome. We analyze the roles of the intrinsic structuring of chromosomes and the forced localization of specific loci, which are observed *in vivo*. Specifically, we examine the segregation pattern of a chromosome that is divided into four structured macrodomains (MDs) and two non-structured regions. We find that strong osmotic-like organizational forces, which stem from the differential condensation levels of the chromosome regions, dictate the cellular disposition of the chromosome. Strikingly, the comparison of our *in silico* results with fluorescent imaging of the chromosome choreography *in vivo* reveals that in the presence of MDs the targeting of the origin and terminus regions to specific positions are sufficient to generate a segregation pattern that is indistinguishable from experimentally observed patterns.

## INTRODUCTION

Storage of genetic information in DNA molecules implies the formation of very long polymers that must be accurately condensed and folded to form functional chromosomes. This organizational challenge has been studied in different bacterial models, yet the molecular basis is still elusive. In bacteria, compaction of the genome due to DNA supercoiling, to interaction with bulk DNA of SMC-like and nucleoid associated proteins (NAPs), and to macromolecular crowding, results in the formation of a structure called the nucleoid (1,2). Faithful transmission of genetic information to daughter cells at each generation requires a timely and spatially controlled segregation process that specifically shapes this nucleoid by positioning the chromosome in the cellular space. According to the bacterial species, the chromosome can adopt different dispositions in the cell (3): in several species, it shows a longitudinal organization along the *oriC-dif* axis (4), while a transversal organization has been reported in *Escherichia coli*.

Different mechanisms, which vary significantly from species to species, can promote chromosome segregation. A spindle-like apparatus composed of a walker type ATPase (ParA) and a DNA binding protein (ParB) that targets a specific sequence (*parS*) has been found in several bacteria (*Vibrio cholera*, *Caulobacter crescentus*, *Bacillus subtilis* and *Streptococcus pneumonia*) (5–7). It was also demonstrated in *C. crescentus* that parS/ParB contributes to chromosome organization (4). The additional recruitment of condensin-like proteins to the origin region by the ParB–*parS* complex contributes to chromosome segregation (7–9). In *E. coli*, such a mitotic-like apparatus has not been found, and different hypotheses have been proposed to account for chromosome segregation. First, it was proposed that the energy that is required for initial chromosome segregation could arise from the replication process that occurs at mid-cell in a static replication factory (10). However, in *E. coli*, the two replication forks follow the chromosomal arms. The capture extrusion model, which was originally proposed for *B. subtilis* (11) is hence irrelevant for this bacterium (12,13). More recently, it was proposed that a loss of cohesion between replicated origin regions could trigger global chromosome movement and mediates chromosome segregation (12). It has also been suggested that entropic exclusion of replicated chromosomes might participate in the segregation process (14) (see below). A number of other recent studies on *E. coli* have noted key proteins that are required for segregation to properly occur. These proteins include the topoisomerase IV (15), the MukBEF condensin (16) and the MatP structuring factor that binds the Ter macrodomain (MD) (17,18). However, the influence of these proteins on chromosome segregation might be indirect, because these proteins are enzymes or structuring factors that favor condensation of the chromosome.

In a pioneering study in 2006, Jun and Mulder (14) applied polymer physics concepts to the bacterial chromosome. They argued that confinement would favor the segregation of chromosomes by the sole force of entropy. On the basis of chromosome models consisting of ∼80 nm structural subunits (14,19,20), this scenario was corroborated in the case of two fully replicated chromosomes. The efficient unmixing of replicating chromosomes was also confirmed by polymer simulations in a situation where newly replicated DNA would follow a specific pathway on the edge of the nucleoid (14,21). However, the formation of MDs is difficult to reconcile with an entropy-based structuration of chromosomes (22,23). Moreover, recent experiments revealed specific, i.e. non-homogeneous, structural features of chromosomes at the cellular scale (24,25,26). Additional mechanisms are thus expected for chromosome organization and chromosome segregation to occur properly.

Our goal in this study is to deepen our understanding of the influence of entropy on bacterial segregation. To this end, we perform numerical simulations of a polymer model of the *E. coli* chromosome using experimentally determined confinement parameters. Our methodology consists in testing models of minimal complexity that can capture *in vivo* features. We thus integrate as few (known) biological parameters as possible and, using indicators that assess the quality of the *in silico* segregation, we compare the results of every simulation with the experimental patterns. Our findings suggest that entropy by itself fails to drive chromosome demixing and fails to promote a correct chromosome disposition. Interestingly, adding only a few experimentally observed constraints to the polymer model dramatically increases the efficiency of the entropy-driven segregation. Specifically, the targeting of the origin and terminus regions to specific positions and the folding of the polymer into MD-like domains is sufficient to generate a segregation pattern that is indistinguishable from the experimentally observed pattern.

## MATERIALS AND METHODS

### Chromosome models

**Figure 1.**
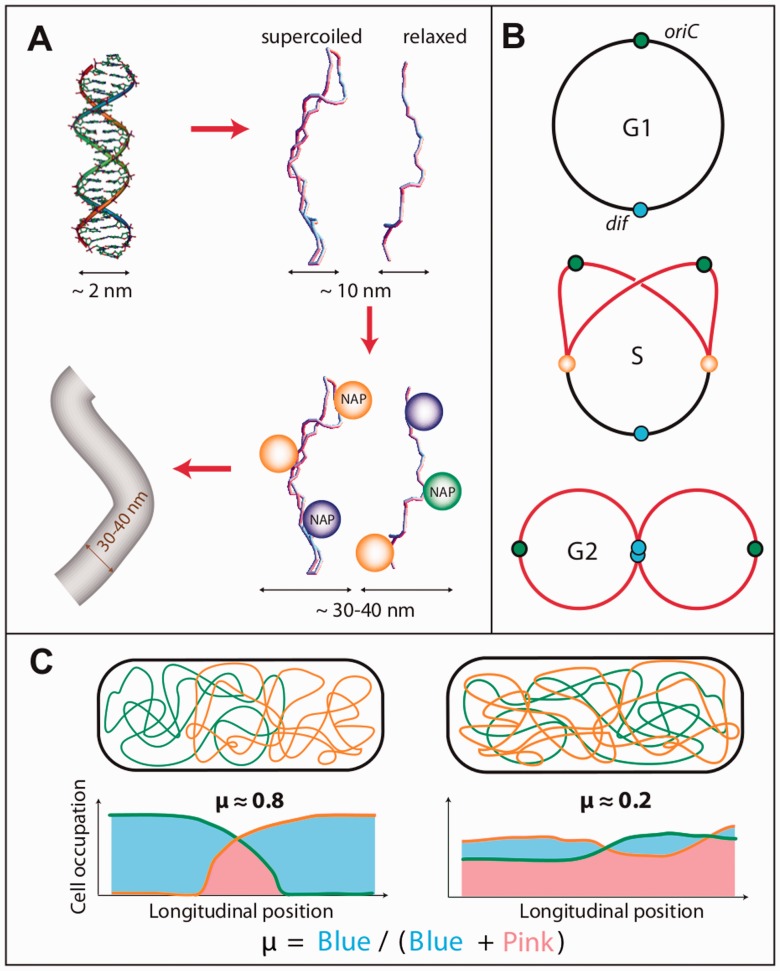
Polymer model of the bacterial chromosome. **(A)** A fiber model of bacterial DNA corresponding to a coarse-grained description of NAP-coated DNA. Naked DNA is a fluctuating polymer that can form plectonemic structures *in vivo* due to the presence of supercoiling. The effective diameter of DNA bound by NAP can be as large as 40 nm (29). **(B)** We consider (i) a single circular chromosome (*E. coli* genome) for the G1 phase, (ii) three long chromosome pieces connected to each other by the replisomes (orange spheres) for the S phase (replicated chromosomes in red) and (iii) two circular chromosomes that are associated together at the replication termini (blue spheres) for the G2 phase. **(C)** The unmixing parameter μ is defined as the absolute difference (blue areas) between the mass distribution of the chromosomes along the nucleoid length (longitudinal position). The values in the figure are indicative.

**Figure 2.**
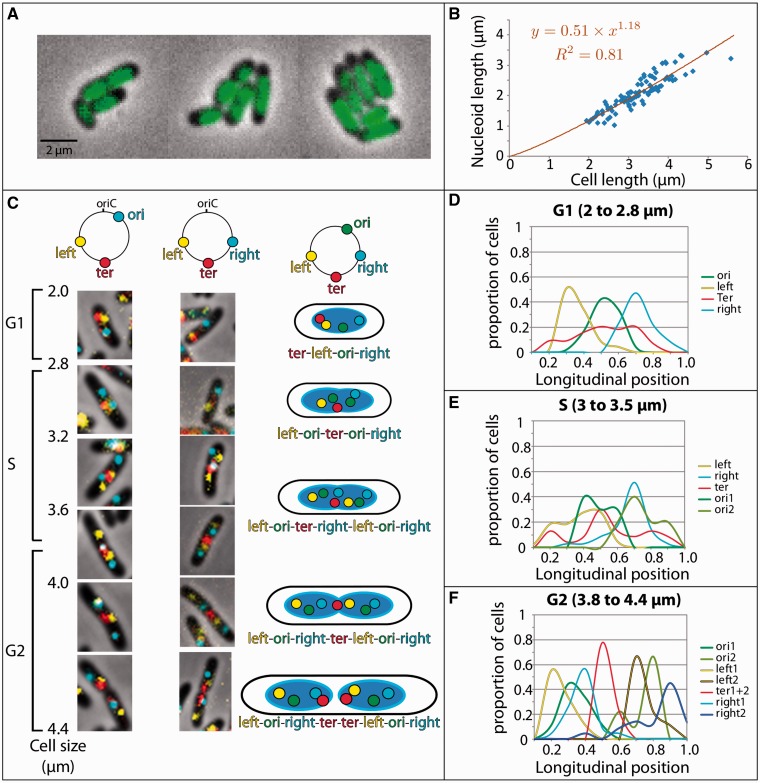
*In vivo* organization of the *E. coli* chromosome*.* **(A)** Imaging of a nucleoid stained with DAPI in *E. coli* cells that were grown in minimal medium A supplemented with glycerol. The nucleoid staining occupies ∼40–50% of the cell volume. DNA free zones are observed at the poles (35% of the cell length) and on the lateral edges (18% of the cytoplasm diameter). **(B)** Nucleoid length as a function of the cell length [103 cells imaged as in A are reported (blue diamonds)]. The lengths were measured using the linescan function of the Image J software. The full width at half maximum was recorded for the DAPI and phase contrast signals. On average, the ratio of the nucleoid length to the cell length is 0.61 ± 0.04. **(C)** Spatial localization of *parS*/ParB^P1^ (cyan), *parS*/ParB^pMT1^ (yellow) and MatP-mCherry (red) in *E. coli* cells grown in minimal medium A supplemented with glycerol. The *parS*/ParB^pMT1^ tag was located in the left replichore at 2 616 013 bp from *thr* (left region). The *parS*/ParB^P1^ tag was located in the right replichore either at 4 413 507 bp (ori region, left imaging panels) or at 738 100 bp (right region, right imaging panels). Imaging panels are arranged from top to bottom from the smallest cells (newborn cells) to the largest cells (onset of division). Each image is representative of the most frequently observed pattern, which is schematically indicated on the right column. **(D–F)** Longitudinal positions with respect to the cell length of the ori, left, right and ter foci in the G1 (D), S (E) and G2 (F) phases. Cells were orientated according to the most polar left focus. In the G1 phase, the ter–left–right and ter–right–left patterns were considered to be equivalent; in the late S and G2 phases, the left–right–ter–right–left pattern (observed in 63% of the population) was differentiated from the left–right–ter–right–left pattern (37% of the population). For each strain, foci positioning was automatically analyzed on 500 hundred cells with the microbetracker software (30) and custom Matlab M-files (31). The length of the S phase and the timing of replication initiation were determined by imaging the SSB-YPet fusion as described in (13,18).

We simulated *E. coli* chromosomes using a worm-like chain (WLC) model (27,28), i.e. a flexible fiber model, of the bacterial chromosome (Figure 1; Supplementary Figure S1). Chromosomes were embedded in a volume whose dimensions corresponded to the nucleoid that is observed *in vivo*: a diameter equal to 800 nm and a length varying from 1.8 μm (G1 phase) to 3.6 μm (G2 phase) (Figure 2). We excluded the possibility of the fibers to overlap (self-avoidance constraint) by using hard-core diameters between 25 and 50 nm [∼40 nm thick nucleoprotein fibers have been observed in the case of rapidly diving cells (29)]. Our findings being qualitatively insensitive to the exact value of the diameters, we report results obtained with a 35-nm thick fiber. The base-pair density along the fiber was fixed at 100 bp/nm and the persistence length at 100 nm, a value that is much smaller than the diameter of the nucleoid.

In the G1 phase, we simulated a single 4.6 Mb long circular chromosome. In the S phase, we mimicked a halfway replicated chromosome by simulating three 2.3 Mb long linear polymers whose extremes were bound together (Figure 1B; Supplementary Methods). In the G2 phase, we simulated two 4.6 Mb circular chromosomes and imposed the termini of replication to remain bound together.

### Macrodomain condensation modeling

The condensation of a genomic region into a single MD with center *c* was modeled by constraining the genomic loci to remain within a sphere of center *c* and diameter equal to 360 nm (lower bound of the estimated size of MDs *in vivo*) (Supplementary Methods). The estimation of the size of the MDs that are imaged *in vivo* was based on the plateau that is observed for the mean-square displacement (MSD) measurements for long time intervals along the lateral (⊥) and longitudinal (∥) directions of the cell (35). We found 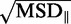 = 220 nm and 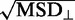 = 240 nm for Ter, 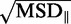 = 400 nm and 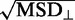 = 280 nm for Ori, which respectively correspond to gyration radii on the order of 200 and 300 nm, and hence, to diameters on the order of 400 and 600 nm, respectively.

### Simulations and thermodynamic analysis of the cellular organization of chromosomes

Off-lattice self-avoiding WLC models were simulated in an implicit nucleoid solvent, i.e. in a continuum medium that is characterized by its thermal energy *k_B_T*, with *k_B_* the Boltzmann constant and *T* = 310 K the physiological temperature. Each chromosome consisted of a semi-flexible polymer composed of a succession of *N* impenetrable cylinders (three cylinders per persistence length)—*N* = 1392 for the 4.6 Mb circular chromosomes. The state space of our polymer models was sampled using a standard Monte–Carlo procedure (Metropolis accept/rejection rule), which guarantees reaching thermodynamic equilibrium at sufficiently large time if ergodicity is not broken (32). Note that not only the formation of the nucleoid is beyond the scope of our analysis but, because it would require integrating explicitly the nucleoplasm together with a model of supercoiled DNA (33), it is also beyond the capacities of current simulation techniques.

The initial polymer conformations were obtained by first equilibrating the system in the presence of all forces and without confinement (large initial embedding volume). The cell volume was next slowly reduced down to the nucleoid volume, and the thermodynamic properties were eventually computed. The slow reduction of the embedding volume aimed at ensuring that the conformations were the most likely from a thermodynamic point of view (by preventing as much as possible the formation of long-living metastable kinetic conformations); the initial large volume prevented chromosome pieces from becoming trapped inside MDs during their condensation.

For each set of parameters (condensation potentials, localization forces, see Supplementary Methods), we performed two simulations. Each simulation consisted of two stages: (i) an initialization stage (see above) and (ii) a thermodynamic analysis. The thermodynamic analysis was realized for the three phases by running two long simulations containing 10^9^ (G1), 1.5 × 10^9^ (S) and 2 × 10^9^ (G2) time steps—the simulation time unit is the ‘sweep’, which corresponds to a Monte–Carlo updating of one random set of contiguous cylinders. When the two simulations were incompatible (because of the possible existence of different metastable states), we ran two additional simulations to test the possibility of new metastable states. In all cases, thermodynamic properties were computed by considering the trajectories that were the most similar to the cellular organization that is observed *in vivo* or, in the absence of any similarity, by considering the first pair of trajectories. Simulations were performed using the computing facilities at the CRG in Barcelona (Spain), the ISC-PIF Île-de-France in Paris (France) and the Institute for Synthetic and System Biology in Evry (France).

### The unmixing parameter μ

μ highlights the difference of the mass distribution of the chromosomes along the longitudinal length (Figure 1C). To determine it, we divide the nucleoid volume into 200-nm-thick slices (*s_i_*) and compute the normalized histogram *h*(*s_i_*) for the presence of monomers. Given two chromosomes *c*1 and *c*2, we thus define 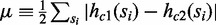. For a given simulation, the distribution of the values taken by 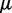 was computed by considering one conformation every 10^5^ (leading to more than 10^4^ different conformations, depending on the replication stage).

## RESULTS

### *In vivo* analysis of chromosome organization

We first determined *in vivo* the parameters that govern the DNA confinement under conditions in which *E. coli* cells have a low chromosome complexity. In slow growth conditions, *E. coli* performs a eukaryotic-like cell cycle with G1, S (DNA synthesis) and G2 phases. Replication of the chromosome is initiated and completed during the same cell cycle; thus, the cells contain between one and two copies of the chromosome. The cell lengths range from ∼2 µm at birth to ∼4.6 µm at the moment of division (Figure 2A and B), and the cell diameter is constant at ∼1 µm (900 nm for the cytoplasm). The length of the nucleoid is approximately proportional to the cell size (Figure 2B), which suggests that DNA confinement is constant throughout the cell cycle.

In *E. coli*, segregation is initiated progressively during replication (34). We used *parS* tags and MatP-mCherry fusion to localize different regions of the chromosome (ori, left, right and ter) and to observe their subcellular localization in cells of various ages during the cell cycle (Figure 2C)—in the following sections, the tags will also refer to the chromosome regions themselves; note that Ori, Ter, Right and Left (starting with a capital) will indicate that the corresponding region (ori, ter, right or left) is folded into a MD (see below). The simultaneous observation of two regions in combination with ter allowed us to accurately map the respective positioning of each region of the chromosome (Figure 2C–F). As reported in previous studies (12,34–36), each tag presented a specific localization pattern during the cell cycle. The replication was monitored under the same conditions by imaging an SSB-YPet fusion.

Figure 2C shows the most frequent cell types that can be observed in the population (frequency >50%). The cartoons on the rightmost column illustrate the estimated localization of ori, right, left and ter in a single cell in the G1, S and G2 phases and at the onset of division; below, we use the G1, S and G2 phases to test the quality of the localization patterns that are obtained *in silico*. During the G1 phase (cell sizes from 2 to 2.8 µm), cells present one focus of each tag (∼10% of the cells presented two ter foci) and an orientation according to the left focus reveals a distinct left–ori–right pattern with the ter region that is localized either at one pole or at mid-cell. In the S phase, we analyzed cells containing two ori foci, one left (or one right) and one ter focus (cell size from 3 to 3.5 µm). Our imaging reveals a localization of the sister ori tags (presumably migrating to the cell quarters) in between mid-cell and quarter positions where they remain located until the end of the cell cycle. We also observe a relocalization of the left, right and ter tags toward the mid-cell. Then, the left and right tags duplicate (∼3.6 µm, end of the S phase) as monitored by the disappearance of the SSB-YPet focus. In the G2 phase, we observe a distinct left–ori–right–ter–left–ori–right pattern that is followed by a duplication of the MatP-mCherry focus (ter region) at the onset of division (4–4.6 µm). In the largest cells, the two replicated sister ter regions remain associated for several minutes (G2 phase) before they split.

These observations allowed us to define the three predominant longitudinal patterns for the organization of the chromosome during the cell cycle: ter–left–ori–right (G1), ori1–left–ter–right–ori2 (S) and left–ori–right–ter–ter–left–ori–right (G2) (Figure 2D–F).

### *In silico* analysis of chromosome organization

We performed numerical simulations of a polymer physics model (Figure 1) to question the mixing properties of the *E. coli* chromosomes. The chromosomes were modeled as flexible worm-like chains (WLC) that cannot overlap (self-avoidance effect). The WLC model provides a coarse-grained description of protein-coated DNA (Figure 1A) that is simple enough so that, using Monte–Carlo simulations, it can be used to investigate the folding properties of long biomolecules (‘Materials and Methods’ section). The embedding volume that contains the chromosome(s) was considered to be that of the nucleoid with a diameter equal to 800 nm and a length that varies during the cell cycle (see below).

Our simulations aimed at determining the most likely cellular organizations that are expected from a thermodynamic point of view for a given set of constraints at a given time of the cell cycle (e.g. by imposing the localization of specific loci). To accomplish this goal, the polymer chains were discretized into cylinders, and motions of randomly drawn blocks of contiguous cylinders were successively proposed. Provided that these motions did not lead to self-overlapping, they were accepted according to microscopically reversible transition rates that depend on the variation of the energy of the polymer and on the available thermal energy (*k_B_T*) coming from the cytoplasm, with *k_B_* the Boltzmann constant and *T* = 310 K the physiological temperature. The polymer energy always included the bending energy of the chain. Depending on the additional constraints, it further included the condensation potentials for the MD structuring and the energies associated with localization forces (Supplementary Figure S1).

We report the cellular organization of three different chromosome states (Figure 1B). Each system reflects the status of one phase, namely, (i) a single circular chromosome for the G1 phase, (ii) three long chromosome pieces connected by the replisomes for the S phase, one piece corresponding to the unreplicated DNA plus two pieces corresponding to the replicated DNA (replisomes were not explicitly considered in the simulations) and (iii) two circular chromosomes connected together at the replication terminus for the G2 phase. The gyration radius of the unreplicated circular chromosome is 3.2 times larger than the nucleoid radius which corresponds to the case of weak confinement (Supplementary Figure S2). The nucleoid lengths for the three phases were taken to be equal to (i) 1.8 and 2 µm, (ii) 2.6 and 3 µm and (iii) 3.6 µm, which correspond to the upper lengths that are measured *in vivo* (Figure 2B). Chromosomes with dissociated termini were only observed in a very small percentage of cells; we thus analyzed this system only by comparison to the G1 phase (Supplementary Figure S3, see below). The replication stage in the S phase was considered to be at midway of the replichores, at the upper borders of the left and right MDs (Left and Right) (37).

**Figure 3.**
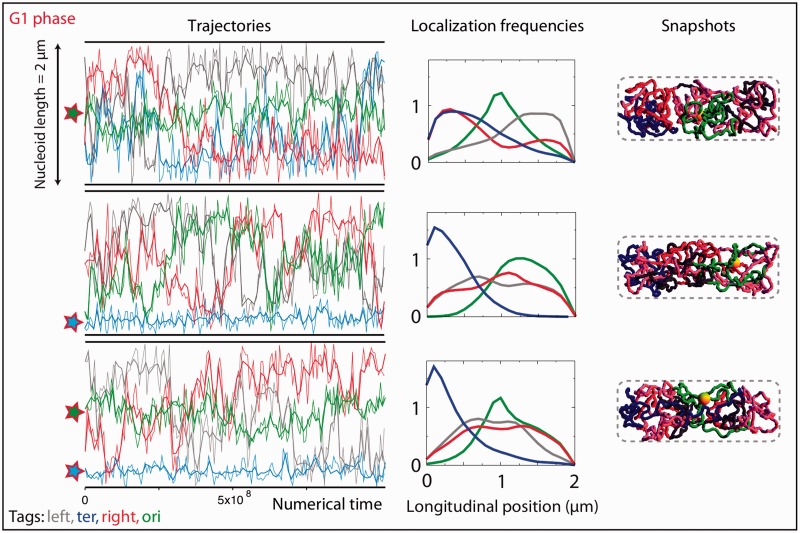
Modeling the G1 phase with homogeneous chromosome polymers; impact of a forced localization of the origin and terminus of replication. Left column: trajectories obtained by simulating the folding of a homogeneous 4.6 Mb long chromosome. Every curve shows the trajectory of a locus that is located in the center of the ori (green), left (gray), right (red) and ter (dark blue) regions; note that, for the sake of visibility, in this figure and in the following ones we use colors that are different from those of the fluorescent tags (Figure 2). The *y*-axis represents the position along the nucleoid; the *x*-axis represents the numerical time. The thin traces represent the trajectories of a subset of points. The thick traces are Bezier interpolation of the raw data. The forced localizations of the origin and/or the terminus of the replication are indicated by the green and blue stars, respectively. From top to bottom: forcing the localization of ori at the center of the cell, forcing the ter region at the pole and forcing both ori at the mid-cell and ter at the pole. Center column: normalized histograms for the localization along the nucleoid of each of the four regions (same color as in the left column). Right column: snapshot showing a typical conformation that is visited by the chromosome during the simulation. The regions are painted with their color (see the other columns), the NS regions are painted in pink and the yellow sphere indicates the replisome.

**Figure 4.**
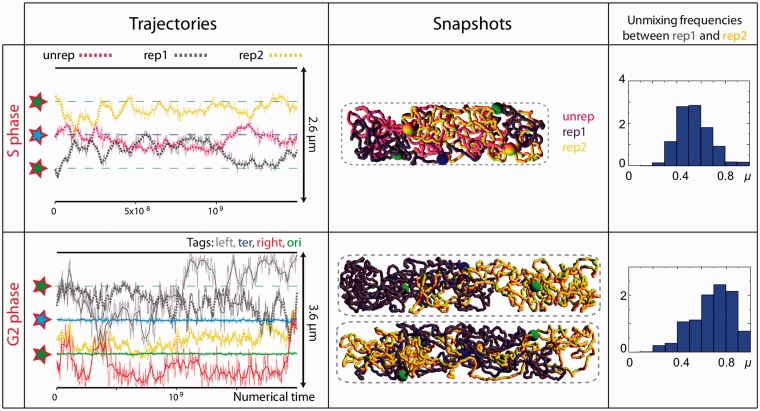
Modeling the S and G2 phases with homogeneous chromosome polymers; impact of a forced localization of the origin and terminus of replication. Left panels: the dotted curves indicate the trajectories of the center of mass of the two replicated chromosomes [rep1 (dark gray) and rep2 (yellow)]. In the S phase, the unreplicated part of the chromosome (unrep) is indicated in pink. In the G2 phase, we report as well the trajectories of the regions belonging to one chromosome (same color code as in Figure 3); the green and blue stars, respectively, indicate the forced localizations of the origin and terminus of the replication (the blue and green dashed lines indicate the corresponding trajectories). Center panels: snapshots showing a typical cellular organization of the chromosomes (same color code as on the left panels). In the G2 phase, the two replicated chromosomes tend to demix (upper snapshot). However, even in a seemingly unmixed situation, pieces of one chromosome can invade the other territory (lower snapshot). This mixing/unmixing profile is confirmed by the distribution of the values of the unmixing parameter (right panels). Right panels: distributions of the values of the unmixing parameter, μ, between the two replicated chromosomes, which were computed by considering more than 10^5^ conformations in each case. The distributions between the replicated chromosomes and the unreplicated chromosome in the S phase are shown in Supplementary Figure S7.

**Figure 5.**
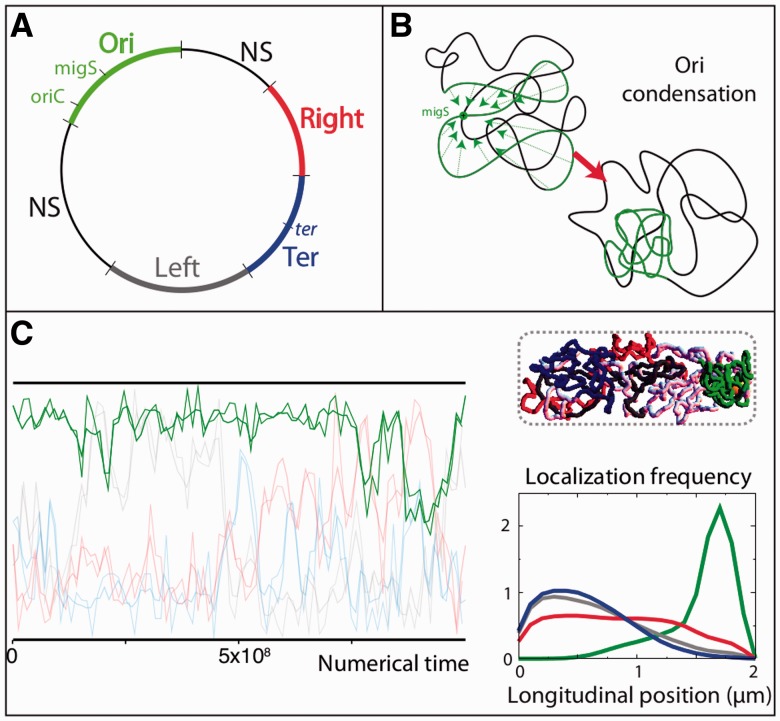
MD-like condensation drives subcellular localization. **(A)** MD organization of the *E. coli* chromosome [adapted from (35)] with the NS domains in black (same color code as in Figure 3). The origin (*oriC*) and terminus (*ter*) of replication, as well as *migS* that is located approximately at the center of Ori, are indicated. **(B)** Schematic representation of the folding of the ori region into a MD. The motion of the Ori loci is biased toward the center of the region (*migS*) by a harmonic potential, leading to a 360-nm diameter MD (Supplementary Methods). **(C)** Left panel: trajectories of the tagged regions for a chromosome containing a single MD (G1 phase). A snapshot of a typical conformation is indicated on the top right panel. The localization diagram (bottom right panel) illustrates the stable polar localization of the MD region.

**Figure 6.**
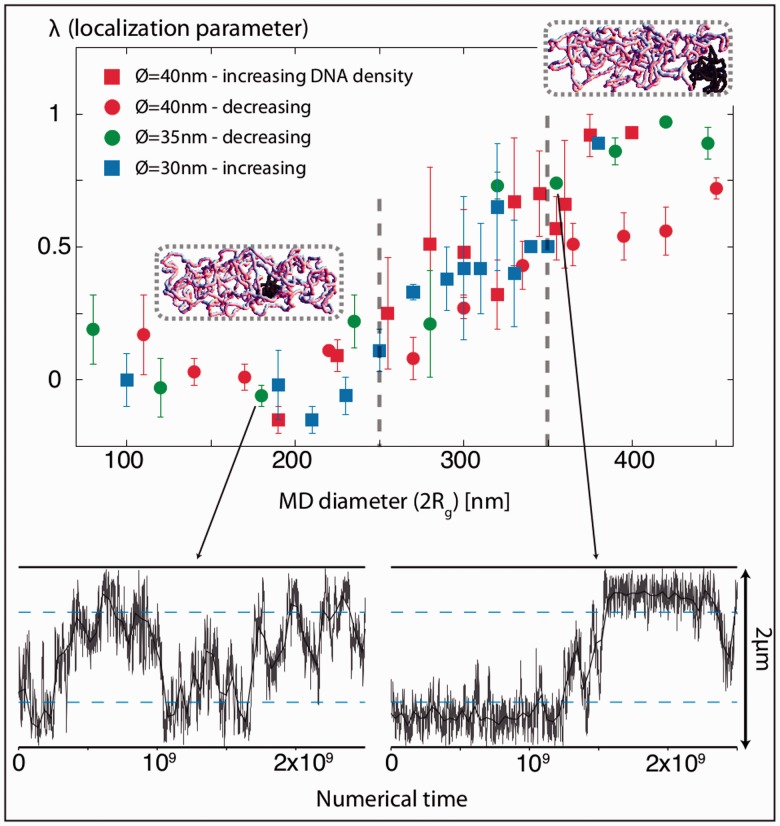
Impact of the structural properties of a MD on its localization. The upper panel indicates in the case of a single MD and a single chromosome (G1 phase) the localization of the MD as a function of its diameter. A value of λ close to 0 reflects a lack of localization specificity. In contrast, λ = 1 reflects a strong localization of the MD at the poles. Four condensation conditions were tested, based on three fiber thicknesses [40 nm (red), 35 nm (green) and 30 nm (blue)] and two condensation protocols [increasing (squares) and decreasing (discs) DNA density]. All the curves show the same characteristics, with poor localization tendency below 250 nm (left dashed line) and strong localization tendency above 350 nm (right dashed line). The chromosome snapshots highlight the typical conformations obtained in each regime (MD in black). The lower panels show the MD trajectories for sizes that are below 250 nm (left panel) and above 350 nm (right panel). The dashed blue lines indicate the cell quarters beyond which the MDs are considered to be at the poles. The error bars in Figure 5D indicate the variance of the results over two 2.5 × 10^9^ sweeps long simulations (‘Materials and Methods’ section).

**Figure 7.**
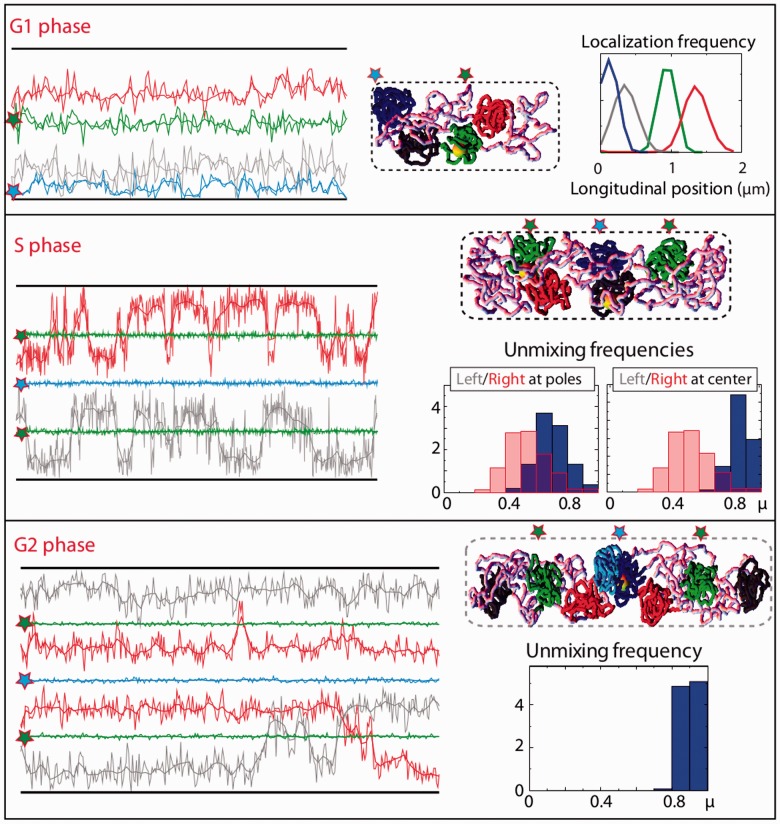
MD structuring and forced localizations of ori and ter recapitulate *in silico* the segregation pattern that is observed *in vivo*. **(A)** In the G1 phase, forcing Ori to be located at the center of the cell and Ter to be located at the pole leads to a very stable positioning of Left and Right on each side of Ori, as illustrated by the trajectories (left panel), the snapshot (center panel) and the localization plot (right panel). **(B)** In the S phase, the cellular organization oscillates between different conformations, with a stable localization of the Left and Right either at the pole or between the quarters and mid-cell (see the trajectories and the snapshot). The distributions of the values of μ (bottom right panels) show that unmixing is dramatically enhanced in this configuration (blue) compared to homogenous polymers (pink). Unmixing is very efficient when Right and Left are observed between the quarters and mid-cell (rightmost panel). **(C)** In the G2 phase, in the presence of MDs, the chromosomes are fully unmixed (μ values peaking at ∼0.9). The forced positioning of the ori and ter leads, in each half of the cell, to an organization that is very similar to the organization that is observed *in vivo* (Left–Ori–Right–Ter, see the trajectories and the snapshot).

Simulations were conducted by first equilibrating the polymers in a large embedding volume. The dimensions of the volume were then progressively reduced down to those of the confining nucleoid. The statistical properties of the polymers were eventually computed in the resulting nucleoid by running two long simulations (‘Materials and Methods’ section). The most likely cellular organizations obtained with the simulations (Figures 3–7) were compared to those obtained with the *in vivo* imaging (Figure 2C–F). To this end, we report in the case of our simulations the trajectories of specific loci located in the center of each region, the trajectories of the centers of mass for both the unreplicated and replicated chromosomes (S and G2 phases) plus a representative snapshot of the chromosome conformation in the nucleoid—see e.g. Figures 3 and 4. In addition, we capture the mixing properties of the chromosomes thanks to a quantitative parameter, μ. This parameter provides the unmixing status between the replicated chromosomes at a given time (Figure 1C). It reflects the difference of the mass distribution of the chromosomes along the longitudinal axis of the nucleoid (‘Materials and Methods’ section) and varies from 0 (completely mixed chromosomes) to 1 (unmixed chromosomes). For every simulation, we report the distribution of more than 10^5^ values of μ, each one of them corresponding to a conformation visited by the chromosomes. We denote 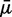 the corresponding average value (one simulation = one value of 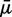).

### Inefficient demixing and incorrect cellular organization of homogenous chromosome polymers

In the absence of any other internal structuring of the chromosomes than the folding of DNA inside the bacterial fiber, our simulations show that, in the G1 phase, no specific localization of any of the chromosome regions can be achieved (Supplementary Figure S4). In the S phase, we find that the two replicating chromosomes tend to mix together (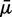 = 0.3–0.5). Moreover, we always observe at least the mixing of two of the three chromosome pieces (Supplementary Figure S5). In the G2 phase, compared to the case of two independent chromosomes, the physical linking of the two chromosomes at the terminus of the replication leads to a slightly more frequent localization of the ter region at mid-cell (Supplementary Figure S2). Although the chromosomes are more efficiently segregated than in the S phase (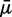 = 0.5–0.7), we observe frequent trapping of one chromosome region inside the other chromosome territory (Supplementary Figure S5). Moreover, no specific localization is observed for the remainder of the chromosome.

Altogether, these results show that the sole polymer entropy is not sufficient to demix replicating chromosomes. They also show that the specific localizations of chromosome regions must be imposed by parameters that are extrinsic to the chromosome.

### Influence of forcing specific localizations for oriC and dif

The most documented features of bacterial chromosome segregation are the choreography of oriC and dif. We thus forced *in silico* the localization of these loci according to their localization *in vivo* (Figure 2) and investigated the resulting mixing properties and the relative positioning of the chromosome regions (results for the G1 phase, respectively, for the S and G2 phases, are shown in Figures 3 and 4 respectively).

In the G1 phase, by imposing an appropriate localization of ori at mid-cell, we find that the left and right regions tend to flank ori in opposite halves, with some preference for the poles, while the ter region mixes together with either the left region or the right region. Next, we observe that forcing the localization of ter at a nucleoid pole drives the remainder of the chromosome away from it, with an ori region that is not specifically localized. Finally, imposing the localizations of both ori and ter does not lead to any specific localization of the left and right regions, which rather mix together.

In the S phase, when the ori localizations are forced to the quarter positions, the ter region tends to locate at the poles and the chromosomes mix to a large extent (Supplementary Figure S6). Mixing properties remain similar when the mid-cell localization of ter is additionally imposed, with the two replicated regions that mix together to a large extent (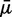 = 0.55–0.65) but also that mix with the non-replicated region (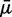 = 0.4–0.75, upper panels in Figure 4). In the G2 phase, the forced localization of ori at the cell quarters induces an efficient demixing (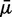 = 0.6–0.8). However, imposing the mid-cell localization of ter tends to favor mixing again with frequent trapping of chromosome regions inside the territory of the other chromosome (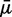 = 0.4–0.8, lower panels in Figure 4). This observation can be explained by the fact that a forced localization also reduces the tendency for the surrounding chromosome region to invade the cellular volume and, hence, to exclude the other chromosome parts. As a consequence, the other chromosome regions can mix together more easily.

These observations do not depend on the microscopic details of the polymer. For instance, although a larger diameter of the fiber tends to stabilize the unmixing state in the G2 phase, which is in agreement with Jun *et al.* analysis of the mixing properties of replicated chromosomes (19–21), chromosomes strongly mix together in the S phase (Supplementary Figure S8). Thus, our results show that the sole polymer entropy and the specific positioning of the ori and ter loci are not sufficient to demix replicating chromosomes.

### A differential level of chromosome condensation leads to strong segregating forces

On a large scale, the *E. coli* chromosome is not linearly organized *in vivo* (Figure 5A). It can be divided into four regions (ori, left, right and ter) that are observed to condense into four distinct MDs (Ori, Left, Right and Ter, respectively), plus two non-structured (NS) regions, which behave differently from the MDs (35,37). MDs present several interesting properties: (i) they are genetically insulated from each other (37), (ii) they occupy a well-defined territory at any stage of the cell cycle and (iii) loci from the MDs present a reduced mobility compared to the NS regions. Ter is the best described MD. Its structuring relies on the binding of the MatP protein to 23 *matS* sites that span the entire MD (17). MatP forms tetramers that bridge two distant *matS* sites (38); it is responsible for its reduced mobility (17) and for its anchoring to the mid-cell (18). In contrast, the structuring mechanisms that are involved for the condensation of Ori, Right or Left are not yet characterized. Nevertheless, for the sake of simplicity, in the simulations we assume that every MD is folded according to the same process (see below).

*In vivo*, the folding of (∼800 kb) genomic regions inside condensed MDs results in enhanced physical contacts between the loci that belong to the same region (36)*. In silico*, this condensation process was modeled by including forces internal to the chromosomes, which biased the motion of genomic loci toward the center of the region (Figure 5B; ‘Materials and Methods’ section). In this context, we considered MDs with a diameter equal to 360 nm, corresponding to a lower bound of their estimated size *in vivo* (‘Materials and Methods’ section). The NS regions were considered to be unconstrained and, therefore, followed the basic polymer properties described in Figure 1.

Strikingly, the presence of a single MD in our polymer models induces a very specific cellular organization of the chromosomes. For example, in the G1 phase, the folding of ori as an MD leads to a localization of the entire ori region at one pole of the nucleoid (Figure 5C). To better understand this phenomenon, we numerically investigated the impact of the properties of the MD on its localization. Our rationale was that, if the MD is so small that it almost coincides with the bacterial fiber, then its impact on the cellular organization should be minor. In contrast, a large and dense MD should behave as a large solid ball. Consequently, an ‘osmotic-like pressure’ coming from the ‘jiggling’ chromosome part should expel the MD toward the pole, just as the motion of microscopic molecules can generate an osmotic pressure on macroscopic objects because of their reluctance to be ‘trapped’ in small volumes [depletion effect (39)]. In this context, we numerically determined the cellular organization of a single circular chromosome containing a single MD whose size and density were controlled (nucleoid length = 2 µm, G1 phase). To test the generality of our results, we investigated three fiber widths (diameters 30, 35 and 40 nm) plus two protocols for the MD condensation (Supplementary Methods).

We characterize the tendency of the MDs to be located at the nucleoid poles by computing a localization parameter 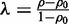. ρ is the frequency (fraction of time) to find the MD at the nucleoid poles, i.e. beyond ¼ and ¾ of the cell length; ρ0 is the corresponding frequency when the size of the MD is equal to the diameter of the fiber (smallest MD). λ close to 0 (ρ = ρ_0_) thus reflects a lack of specificity in the localization of the MD. In contrast, λ = 1 (ρ = 1) reflects a strong localization of the MD at the poles. Within this scope, our simulations reveal the existence of two regimes that are separated by a 250- to 350-nm crossing region, independently of the exact MD composition as well as of the microscopic features of the chromosome fiber (Figure 6). Specifically, MDs with diameters smaller than 250 nm have no specific localization along the cell (λ = 0). In contrast, MDs with diameters larger than 350 nm tend to localize at the poles (λ > 0.5), with the largest MDs presenting the highest λ values. Notably, the typical *in vivo* MD diameters are estimated to be larger than 400 nm (‘Materials and Methods’ section).

### Segregation patterns in the presence of multiple macrodomains

*In silico*, a single MD has a strong impact on the chromosome organization. Could a complete folding of the polymer according to a specific MD organization, as that observed in *E. coli*, be advantageous for demixing and specifically localizing the chromosomes? In the G1 phase (see Supplementary Figure S9), the additional presence of Ter results in a longitudinal organization (Ter-NS-Ori), in which the non-condensed left and right replication arms locate at the center of the cell and Ter and Ori occupy opposite poles. The further presence of Right and Left leads to a very stable pattern (Right/Ter/Left-NS-Ori) in which Right, Left and Ter frequently exchange their positions at one pole. These results indicate that the NS regions play a role in confining Ori to the pole and, more generally, in excluding the MDs from the space they occupy. Interestingly, the Ter-NS-Ori organization is reminiscent of the chromosome organization that has been observed in *C. crescentus* (40) (Supplementary Figure S10), but not of that observed in wild-type *E. coli*. Our findings thus strongly suggest, once again, that the mid-cell localization of Ori must be imposed by a mechanism that is external to the chromosome. Strikingly, when Ori is forced to mid-cell and Ter to one pole, the chromosome switches between two conformations that are observed *in vivo*: Ter–Left–Ori–Right and Ter–Left–Right–Ori. The balance between the two conformations is found to be sensitive to the size of the nucleoid, with a smaller size (e.g. 1.8 μm instead of 2 μm) favoring the Ter–Left–Ori–Right conformation (upper panels in Figure 7).

Note, finally, that *in vivo*, the migration of Ter to mid-cell is delayed until the middle of the S phase. In our simulations, in the absence of a specific Ter localization, Ter flanks Ori at the center of the cell according to a Left–Ter/Ori–Right pattern in the G1 phase (Supplementary Figure S9). Thus, the observed delay for the Ter migration might originate from specific organizational features that are specified by MatP.

### Cell cycle: interplay between macrodomain structuring and imposed locus localizations

To further deepen our understanding of the segregation mechanism during the cell cycle, we investigated the impact of the folding of the different chromosome regions at the different phases (G1, S and G2). The trajectories and snapshots of the typical conformations can be found in Figure 7 as well as in Supplementary Figures S11 and S12 for the S phase and in Supplementary Figure S13 for the G2 phase.

*S phase.* In *E. coli*, segregation is initiated during replication by the migration of newly replicated origins to the cell quarters (corresponding to the mid-cell of the G1 phase). *In silico*, as any folded MD, in absence of any specific localization, in the S phase the Ori’s tend to localize at the poles. Hence, once again, their positioning at the cell quarters must be imposed externally. In this context, in the absence of the folding of the left and right regions, we find that Ter preferentially goes to the poles (Supplementary Figure S11). Thus, its mid-cell positioning, as observed *in vivo*, must be imposed. In the presence of Right and Left, Ter tends to locate preferentially at mid-cell, although it is sometimes found at one pole—one flanking domain (Right or Left) then moves to the center of the cell. Interestingly, this conformation with Ter at one pole and Left (or Right) at the center is observed in 30% of the cells (Supplementary Figure S14). More interestingly, by imposing the replication terminus to be bound at the septum, we find that the chromosome disposition switches between three organizations that, strikingly, match the relative positioning of the pairs of loci *in vivo* (Supplementary Figure S14): « Left–Ori–Ter–Ori–Right », « Ori–Left–Ter–Right–Ori » and « Left–Ori–Ter–Right–Ori » (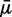 = 0.7, Figure 7). Similarly to the G1 phase, the balance between these three conformations depends on the size of the nucleoid, with a longer nucleoid (e.g. 3 μm instead of 2.6 μm) favoring the Ori–Left–Ter–Right–Ori pattern (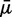 = 1 in this case, Figure 7). Note also that the length of the unconstrained DNA that separates Right/Left from Ter plays a crucial role, with shorter lengths favoring the Ori–Left–Ter–Right–Ori pattern (Supplementary Figure S12).

*G2 phase.* For two fully replicated chromosomes that remain connected together at their replication terminus, the presence of MDs strongly enhances the tendency for chromosomes to demix (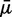 = 0.85–0.9). Once again, the positioning of the Ori’s at the cell quarters must be imposed. In this context, the presence of Left and Right leads to a very stable organization of the type « Left–Ori–Right–Ter–Ter–Left–Ori–Right » (Figure 7, 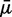 = 0.9), which is a genuine feature of the G2 phase (Figure 2F).

Together with the fact that the numerical outcomes do not depend on the microscopic properties of the polymer models (see Supplementary Figures S15 and S16), these results show that, in the presence of MDs, the specific localization of the origin and terminus of the replication is sufficient to explain the segregation pattern of the *E. coli* cell cycle (recapitulated in Figure 7).

## DISCUSSION

### Simulations under the conditions of *in vivo* imaging

It has been proposed that bacterial chromosomes can demix using entropy as the only driving force (14,21). However, the *in vivo* analysis of whole chromosome folding and segregation revealed complex patterns that were difficult to reconcile with purely entropic behavior (22,23,25,26,41). In this article, we have presented a calibrated quantitative and qualitative approach to monitoring the ability of entropy to segregate chromosomes. We investigated in detail the case of an *E. coli* growing with successive G1, S and G2 phases. We aimed at studying the most likely cellular organization of the chromosomes given a set of internal and external constraints (MD condensation and locus localization, respectively). In this respect, we cannot ensure that the cellular organizations we find are those that will systematically emerge during a dynamic process such as replication. Neither can we ensure that these are the most stable thermodynamical states. Nevertheless, our numerical study shows that the chromosome dispositions that match the *in vivo* patterns, which are obtained in the presence of MDs, are extremely stable. Thus, a quasi-static process in which the internal and external constraints are evolving slowly with respect to the amount of time that is required to locally equilibrate these conformations is likely to allow the chromosome to successively visit these states, hence ensuring a reproducible segregation pattern.

### Mixing properties and specific positioning of homogenous polymer models of chromosomes

In the absence of any other internal structuration of the chromosome than the folding of the DNA inside a thick fiber, we find that fully replicated chromosomes tend to demix, as reported previously (14). However, excursions of one chromosome into the territory of the other chromosome are frequent, as quantified by the values of the unmixing parameter (μ). Moreover, our simulations reveal that chromosomes midway to full replication mix together, which is in disagreement with *in vivo* observations (12), and that targeted localization of the origins or/and the terminus of the replication do not solve the problem. These results, together with recent similar observations about the inefficiency of entropic forces alone to properly demix replicated chromosomes (42), differ from the results reported previously in which segregation was observed throughout the cell cycle (14). A possible explanation is that in Jun and Mulder (14) longer confining volumes were used. More importantly, we believe that the possibility of segregation in Jun and Mulder (14) strongly relied on the proposal that replicated chromosomes, unlike unreplicated DNA, could escape from the nucleoid through a thin outer cylinder. This process would both favor demixing between the replicated and unreplicated chromosomes and enhance the repulsion between the two replicated chains (the repulsion occurs because of the strong lateral confinement). In the absence of any experimental support for this outer cylinder, we did not account for it in this study.

Analysis of FROS tag localization in a number of bacteria always revealed precise targeting of the origin of replication (3,43). In contrast, the stable localization of the origin region to the mid-cell in newborn cells, or to the cell quarters after replication is initiated, was never observed in our simulations. External forces must, hence, impose this specific positioning. Moreover, we did not observe any stable polar localization of the ori region in the newborn cells in the case of a homogenous polymer. In conclusion, neither a transversal *E. coli*-like pattern nor a *C. crescentus* longitudinal pattern was obtained with a simple unconstrained homogeneous polymer model.

### Macrodomain folding and specific targeting: segregation drivers in *E. coli*

Our study reveals that, together with the presence of NS regions, MD-like structuring generate strong segregation forces. In addition, our simulations show that the specific localization of either Ori or Ter or both (depending on the replication stage) is essential to achieve an optimal organization. Thus, just as in the spirit of the recent findings for the large-scale organization of chromosomes in yeast (44,45), most of the organizational features of slow growing *E. coli* chromosomes could be the result of a combination of a few basic physical processes: thermal agitation of the chromosome polymer, condensation of a few chromosome regions and the proper positioning of specific loci such as the origin and terminus of the replication. Notably, the poor segregation obtained by folding the entire chromosome as a string of MDs (absence of NS regions), which lead to entangled sister regions (Supplementary Figure S17), highlights the importance of maintaining two levels of condensation of the polymer. Within this scope, it is important to keep in mind that structural constraints other than *oriC/dif* targeting and MD folding are expected to be involved in the process of chromosome structuration and chromosome segregation (3,46,47). In this perspective, including in the simulations the phenomena of chromatid cohesion (48), replichore asymmetry (36), replisome localization (13), MukB/SMC binding (8) or the transertion of a number of membrane proteins (49), are avenues for future studies.

One important aspect of our findings is the generality of the mechanism. Specifically, the driving forces rely on an osmotic-like pressure for which MDs can be considered as macroscopic beads that are embedded in a jiggling environment that is provided by the NS regions. NS regions can thus be viewed as a loaded entropic spring that pushes the large MDs toward the periphery. In this context, the very nature of the MDs and NS regions should not play any role. The only necessary ingredient is the existence of distinguishable long chromosome regions with different motility properties, which have been observed in *E. coli* (35, 50) or, equivalently, with different levels of compaction as, e.g. in the cellular organization of eukaryotic chromosomes (51).

In accord with recent experimental results about the nature of the driving forces involved in chromosome structuration and segregation in *E. coli* (52), the mechanism we propose here can be described as indirect (23) as it possesses features of both active and passive processes. In this context, the rich diversity of organizations that have been found in bacteria might be the result of slight variations of parameters such as the length of the nucleoid or the size of the NS regions. Interestingly, MD-like domains called ‘topological domains’ (53) or ‘topological associating domains’ (54) have been identified in metazoans. Because the confinement of DNA in the mammalian nucleus is stronger than in *E. coli* (55), we expect these domains to play a crucial role in the overall organization of the nucleus during interphase.

Finally, let us mention that the replication process itself has been shown to introduce an asymmetry in the cellular localization of DNA strands in *E. coli*, with the leading strands localized at the old poles of the cell and the lagging strands near the forming septum (future new poles) (56). As a consequence, in the G2 phase the left (L) and right (R) replication arms are specifically organized according to the sequential order LRLR (36). The strands being indistinguishable in our simulations, we could however not address this problem. The segregation of the *E. coli* chromosome also involves an abrupt separation step: nucleoid splitting (12,57). The existence of this event could not be assessed either in our simulations, for two main reasons. First, understanding the formation and, hence, the splitting of the nucleoid requires to explicitly integrate the surrounding proteins together with a model of supercoiled DNA (33). However, state-of-the-art simulations of the bacterial cytoplasm (58) suggest that this is far beyond of current numerical capacities. Second, we studied the most likely chromosome conformations in a given set of constraints, which included a fixed embedding volume (the nucleoid) for the chromosomes. In this regard, the existence of two very distinct behaviors for the cellular localization of chromosome regions, depending on the size of the nucleoid (Figure 6), could explain how nucleoid splitting appears. The increase of the DNA content relative to the nucleoid space might catalyze such an abrupt step. Alternatively, the nucleoid splitting could result from a rapid folding of a large region of the chromosome (e.g. the ori region) in an MD-like structure.

## SUPPLEMENTARY DATA

Supplementary Data are available at NAR Online. 

Supplementary Data
