# Supplementary material for: Polymer modeling of the E. coli genome reveals the involvement of locus positioning and macrodomain structuring for the control of chromosome conformation and segregation
Source: Nucleic Acids Res. 2013 Nov 3;42(3):1461–73. doi: 10.1093/nar/gkt1005 (PMC3919569; doi:10.1093/nar/gkt1005)
Supplement: Supplementary Data [file supp_42_3_1461__index.html]

Polymer modeling of the E. coli genome reveals the involvement of locus positioning and macrodomain structuring for the control of chromosome conformation and segregation — Polymer modeling of the E. coli genome reveals the involvement of locus positioning and macrodomain structuring for the control of chromosome conformation and segregation — Supplementary Data 

# Polymer modeling of the *E. coli* genome reveals the involvement of locus positioning and macrodomain structuring for the control of chromosome conformation and segregation

## Supplementary Data

files

**Files in this Data Supplement:**

- Supplementary Data - pdf file
- Supplementary Data - pdf file
