# Supplementary material for: Polymer modeling of the E. coli genome reveals the involvement of locus positioning and macrodomain structuring for the control of chromosome conformation and segregation
Source: Nucleic Acids Res. 2013 Nov 3;42(3):1461–73. doi: 10.1093/nar/gkt1005 (PMC3919569; doi:10.1093/nar/gkt1005)

Polymer modelling of the E. coli genome reveals the involvement of locus positioning and macrodomain structuring for the control of chromosome conformation and segregation -- Supplementary Methods

Ivan Junier[1,2]*, Frédéric Boccard[3] and Olivier Espéli[3]*

Modeling localization constrains of chromosomes

In the G1 phase, we simulated the folding of a single circular chromosome whose length = 4.6 Mbs. In the S phase, we used three linear polymers that were bound to each other by pairing the extreme monomers (Figure 1B, main text). We used a harmonic potential $V_S(d) = \frac{k_S}{2} d^2$ that constrained the distance d that separates the pairs of monomers. The potential steepness $k_S = \frac{50^2}{k_B T}$ was set to ensure that the typical distances that separated these monomers did not exceed 50 nm, which is equivalent to a 15-nm fiber-to-fiber distance. We used the same harmonic potential in the G2 phase to impose the termini of replication to remain bound together.

The localization of a specific locus at z_0 along the cell length (axis z in the lower right panel of Supp. Fig. 1) was imposed by including an additional harmonic potential $V_{loc}(z) = \frac{k_{loc}}{2}(z - z_0)^2$, with $k_{loc} = \frac{50^2}{k_B T}$.

Modeling macrodomain condensation

In silico, the *E. coli* MD organization was divided into six 770-kbp-long genomic regions (~ Genome length/6, see Figure 3A in main text). The condensation of a genomic region into a single macrodomain (MD) with center c (*e. g.,* ~ *migS* for the ori region) was modeled by constraining the genomic loci to remain within a sphere of center c and a radius equal to approximately 200 nm. In practice, we used a harmonic potential $V_{MD}(x) = \frac{k_{MDcoli}}{2} x^2$ such that $k_{MDcoli} = \frac{200^2}{k_B T}$, where x indicates the spatial distance between the corresponding locus and the center c. These implementation led to the formation of MDs having a gyration radius equal to ~ 180 nm (diameter = 360 nm). An additional 150-kbp chromosome piece was used to separate Left and Right from Ter.

Cellular organization of a single chromosome in the presence of a single macrodomain (MD)

In order to better understand the capacity of the MDs to drive the overall organization of one or several chromosomes, we numerically investigated in detail the impact of the folding of a single MD. To this end, we varied both the intensity of the condensation potential and the genomic length of the folded region to study the extent to which different MD sizes and different DNA densities within the MD could influence the cellular organization of the chromosome. Specifically, we determined the organizational properties of a single circular chromosome that contained a single MD whose size and density was controlled – the nucleoid length was set to 2µm (~ G1 phase). We investigated three types of fiber widths (diameters 30, 35 and 40 nm), and two protocols for the variation of the potential steepness k_{MD} of the condensing potential (see above) as a function of the genomic size of the domain. As a result, we obtained MDs that have their DNA density that either decreases or increases with respect to the MD diameter (see Figure).

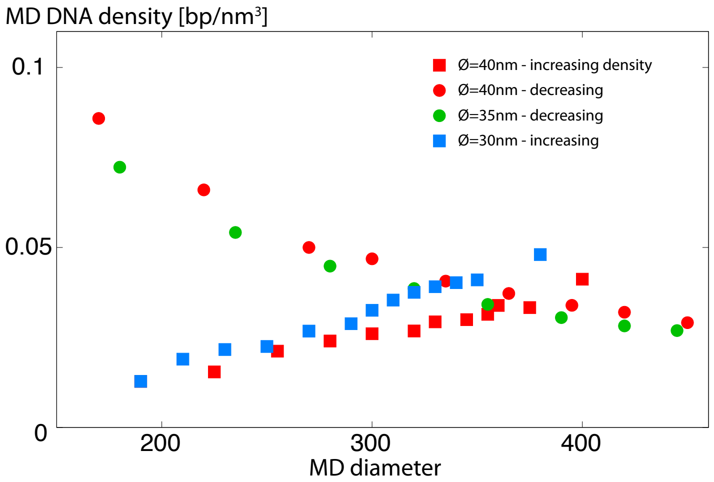

Supplement: Supplementary Data [file supp_gkt1005_nar-02059-f-2013-File009.pdf]
