# Supplementary material for: Polymer modeling of the E. coli genome reveals the involvement of locus positioning and macrodomain structuring for the control of chromosome conformation and segregation
Source: Nucleic Acids Res. 2013 Nov 3;42(3):1461–73. doi: 10.1093/nar/gkt1005 (PMC3919569; doi:10.1093/nar/gkt1005)
Supplement: Supplementary Data [file supp_gkt1005_nar-02059-f-2013-File010.pdf]

Polymer modelling of the E. coli genome reveals the involvement of locus positioning and macrodomain structuring for the control of chromosome conformation and segregation – Supplementary Figures

Ivan Junier[1,2]*, Frédéric Boccard[3] and Olivier Espéli[3]*

In all figures, if not further precised the colors used for the trajectories of the chromosome regions (ori=green, ter=blue, left= gray, right=red) and for the centers of mass of the chromosomes (pink=unreplicated chromosome, yellow and gray = replicated chromosomes) are those used in Fig. 3 and 4 of the main text.

Figure S1

Top panel -- All simulations were based on a worm-like chain model of the bacterial chromosome. The first basic feature of this model is the energy cost (E_b) that the polymer must pay to the solvent (the cytoplasm) to bend – bending here corresponds to the variation of the tangent vector $\vec{t}$ along the curvilinear abscissa of the polymer (s in the figure). The persistence length l_p, which corresponds to the typical length beyond which the polymer looses memory of its directional order, is proportional to the bending modulus (K) and is inversely proportional to the thermal activation: $l_p = K/k_BT$. The second basic feature is the confinement of the chromosomes inside the nucleoid. *Bottom left panel* -- In addition to these basic features, we add condensation forces that allow to fold regions of the chromosomes into macrodomains (MD). *Bottom right panel* -- We also add potentials (V_{loc}) that allow to impose the localization of specific loci (as e.g. the red locus here).

Figure S2

Typical conformations of the chromosomes obtained in the simulations in the absence of confinement, with the green spheres indicating the diameter of the nucleoid that is used in the simulations (800 nm). At stage G1, the radius of gyration is equal to ~ 1300 nm, which is equal to 3.25 times the radius of the nucleoid in the simualtions (400 nm). This corresponds to the case of weak confinement.

Figure S3

Cellular organizations of homogenous chromosome polymers in the ***G2 phase***. The top cartoons represent two types of chromosome constraints: i) with cohesive termini (ter regions bound together, left cartoon) and ii) with separated termini (independent chromosomes, right cartoon). The red curve corresponds to the trajectory of the terminii of the replicated chromosomes when they are bound together. The blue and green curves correspond to the distinct trajectories of the terminii when the chromosomes are independent. The corresponding frequencies for the localization of every region along the nucleoid are plotted in the bottom left panel. The distribution of the unmixing parameter μ (bottom right panel) does not reveal any qualitative difference for the mixing properties between the two cases.

Figure S4

Organization of homogeneous chromosome polymers in absence of any specific locus localization in the G1 phase. *Left panel* – Trajectories of four loci located in the center of the ori (green), left (gray), right (red) and ter (dark blue). One can see that the regions explore the entire cell length during the simulation. Moreover, different simulations lead to different trajectories (not shown). *Center panel* – Snapshot of a typical chromosome conformation; the surrounding dashed box indicates the embedding volume (nucleoid). *Right panel* – Localization plot showing the average localization density of the regions along the nucleoid during a single simulation.

Figure S5

Organization and demixing tendencies of homogeneous chromosome polymers in absence of any specific locus localization in the S and G2 phases. This figure is the counterpart of the main Fig. 4 in which the localizations of ori and ter are forced. *Left panels:* the dotted curves indicate the trajectories of the center of mass of the two replicated chromosomes (rep1 (dark grey) and rep2 (yellow)). In the S phase, the unreplicated part of the chromosome (unrep) is indicated in pink. For this phase, we also show the trajectories of the different tagged regions (lower panel). *Center panels:* snapshots showing the typical cellular organization of the chromosomes; the different chromosomes, i.e. the replicated and unreplicated ones, are painted with the same color as on the left panels. In the G2 phase, the two replicated chromosomes tend to demix (upper snapshot). However, pieces of one chromosome often invade the other territory (lower snapshot). This mixing/unmixing profile is confirmed by the distributions of the values of the unmixing parameter (right panels). *Right panels:* distributions of the values of the unmixing parameter, μ, between the two replicated chromosomes, which were computed by considering more than 10^5 conformations in each case.

Figure S6

Impact of a forced localization of the origins of replication on the demixing properties in the S phase.

The figures show the results of numerical simulations in a situation where ori1 and ori2 are forced to be localized at the cell quarters. *Left panel* – The trajectories are those of the two replicated ori regions (green) that are anchored at the cell quarters (green star) and of the unreplicated ter (blue), left (gray) and right (red). The dashed curves represent the centers of mass of the chromosomes (pink curve for the unreplicated chromosome and yellow and gray curves for the replicated ones). We see that the unreplicated chromosome often mixes together with one replicated chromosome, which is confirmed by the distribution of the μ values. *Right panels* – Distributions of the values of the unmixing parameter, μ, between the three pairs of chromosomes that can be considered using the two replicated chromosomes (rep1 and rep2) and the unreplicated chromosome (unrep). The distributions were computed by considering more than 10^5 conformations in each case.

Figure S7

Impact of a forced localization of both the origin and terminus of replication on the demixing properties in the S phase. The figure shows the distributions of the values of the unmixing parameter, μ, between the three pairs of chromosomes that can be considered using the two replicated chromosomes (rep1 and rep2) and the unreplicated chromosome (unrep). The distributions were computed by considering more than 10^5 conformations in each case.

Figure S8

Upper panel – The left column show the trajectories of the ter, left, right and ori regions in the ***G2 phase*** when ori and ter are specifically localized (indicated by the stars), for homogenous chromosomes, for different parameters of the chromosome fiber (diameter ⌀ and persistnce length lp). The right histograms indicate the corresponding distribution of the unmixing parameter μ. One can see that unmixing is all the more stabilized that the diameter of the fiber is large (e.g. for ⌀=50nm). Nevertheless, even in this case, starting from an unmixed situation, the chromosomes may be found after some time in a fully mixed state as shown at the bottom (a typical conformation of the mixed chromosomes is indicated on the right). *Lower panel* – Trajectories of the ter, left, right and ori regions, histograms of μ between the replicated chromosomes and typical chromosome conformations in the ***S phase***, for ⌀=50nm, lp=100 nm. In contrast to the G2 phase, chromosomes strongly mix together, eventhough the ter and ori regions are specifically localized (stars).

Figure S9

MD structuring imposes strong organizational constraints on a chromosome polymer. The trajectory of four loci located in the center of the ori (green), left (gray), right (red) and ter (blue) regions in a 2 μm long nucleoid (G1 phase). Folded regions in the MDs are indicated with a filled hexagon. From top to bottom: Ori and Ter condensation; condensation of the four MDs (Ori, Ter, Left and Right); four MD condensations plus forced localizations of Ori; four MD condensations plus forced localization of Ori and Ter; four MD condensations plus forced localization of Ori and Ter in a smaller nucleoid (length = 1.8 μm).

Figure S10

Frequency of the localization along the nuecloid of three markers belonging to either the left (top) or right (bottom) arm in the ***G1 phase*** in the presence of Ori and Ter. One can see that chromosomal loci tend to be organized along the cell according to their position along the replichores, as observed in *C. crescentus* (Viollier et al, 2004).

Figure S11

Trajectories of the ter, left, right and ori regions in the ***S phase*** when the macrodomains Ori (green hexagon) and Ter (blue hexagon) are folded. Upper panel: no forced localization. Lower panel: the ori1 and ori2 are forced to locate at the cell quarters (green stars).

Figure S12

Trajectories of the ter, left, right and ori regions in the ***S phase*** taking into account the MD organization of *E. coli*. **A)** *No specific localization is imposed*. In this case, we observe that the nucleoid poles are always occupied by one of the MDs and that the MDs exchange often their positions. **B)** *Forced localization of the Ori's at the cell quarters (green stars)*. In this case, the unreplicated chromosome adopts a Left-Ter-Right organization, whose positioning with respect to the middle of the cell can be shifted. In this simulation, Ter is found at midcell and Right (or Left) at the poles. In the panels CDE, we analyze the situation when the Ori's are anchored to the cell quarters (green stars) and Ter to mid-cell (blue star). We study the impact of two parameters on the cellular organization of the chromosomes: i) the length of the DNA that links Ter to its flanking MDs (Right and Left) and ii) the size of the nucleoid. **C)** short linkers (30 kb) in a 2.6 μm long nucleoid. **D)** long linkers (150 kb) in a 2.6 μm long nucleoid. **E)** long linkers (150 kbp) in a 3 μm long nucleoid.

Figure S13

G2 phase. Two qualitatively different sets of trajectories for ter, left, right and ori in the presence of Ori and Ter (hexagons). Ori1 and Ori2 are anchored to the cell quarters (green stars) and Ter is anchored to mid-cell (blue star). The dashed and full curves are used to distinguish the trajectories of the two replicated chromosomes (rep1 and rep2). The upper panel shows that even in the case of a good segregation, the Ter flanking regions (left and right) tend to mix together. The lower panel shows that these not only mix together but also sometimes enter the territory of the second chromosome.

Figure S14

During the **S phase**, the organization of the chromosome may fluctuate. In this regard, we observe four patterns of cells containing two ori foci (cyan), one left focus (yellow) and one (or two) ter focus (red). The percentage of cells in the population corresponding to each pattern is indicated (we counted 100 cells containing two ori foci and one left focus). The cells containing two ter foci correspond to replicating Ter's (Espeli et al., 2012).

Figure S15

The left panels indicate the trajectories of the ter, left, right and ori regions in the ***G2 phase*** in the presence of MDs when Ori and Ter are specifically localized (indicated by the stars), for different parameters of the chromosome fiber (diameter ⌀ and persistnce length lp). The right histograms indicate the corresponding distribution of the unmixing parameter μ. We see that the chromosomes tend to demix, independently of the precise value of the parameters.

Figure S16

Trajectories of the ter, left, right and ori regions, typical chromosome conformations and histograms of μ between the replicated chromosomes in the ***S phase***, for ⌀=50nm, lp=100 nm, in the presence of MDs. Two results are shown, which differ from the length of DNA that is used to separate Ter from Left and Right (linkers). For short linkers

(bottom panels), Left and Right tend to remain spatially close to Ter, hence stabilizing the unmixing status of the replicated chromosomes.

Figure S17

Left column – Cellular organization in the **S phase** when every region of the chromosomes, i.e. including the non-structured domains (grey and orange hexagons), is folded into a MD and when Ter and Ori are forced to be localized at mid-cell and at the cell quarters, respectively - a 150 kbp free linker DNA was inserted between each pair of two consecutive MDs (cartoon in the left column). *Center column* – The top trajectories show the unreplicated Left, Right and Ter and the replicated Ori's. The bottom trajectories show only the replicated Ori's and NS's (two colors, gray and orange, are used to distinguish the two replicated chromosomes). *Right column* – The top panel shows that compared to the case with the WT *E. coli* MD topography (see Figure 7 in main text) Left and Right are not sharply distributed on each side of Ori. The bottom left panel shows the distribution of the values of the unmixing parameter between the two replicated chromosomes (rep1 and rep2). The values are quite similar to the case of the *E. coli* MD organization (Figure 7 in main text). However, the typical conformations of the chromosomes (snapshot on the right) reveal a systematic intermingled organization where the folded NS regions are respectively localized according to a NS1-NS2-NS1-NS2 pattern (NS1 for rep1 and NS2 for rep2). This intermingled conformation, which does not correspond to a good segregation pattern, leads to a large value of μ because of a strong compartmentalization of the chromosomes (see definition of μ in Fig 1C, main text).

References

Espeli O, Borne R, Dupaigne P, Thiel A, Gigant E, Mercier R, Boccard F (2012) A MatP-divisome interaction coordinates chromosome segregation with cell division in E. coli. *EMBO J* **31:** 3198-3211

Viollier PH, Thanbichler M, McGrath PT, West L, Meewan M, McAdams HH, Shapiro L (2004) Rapid and sequential movement of individual chromosomal loci to specific subcellular locations during bacterial DNA replication. *Proc Natl Acad Sci U S A* **101**(25)**:** 9257-9262

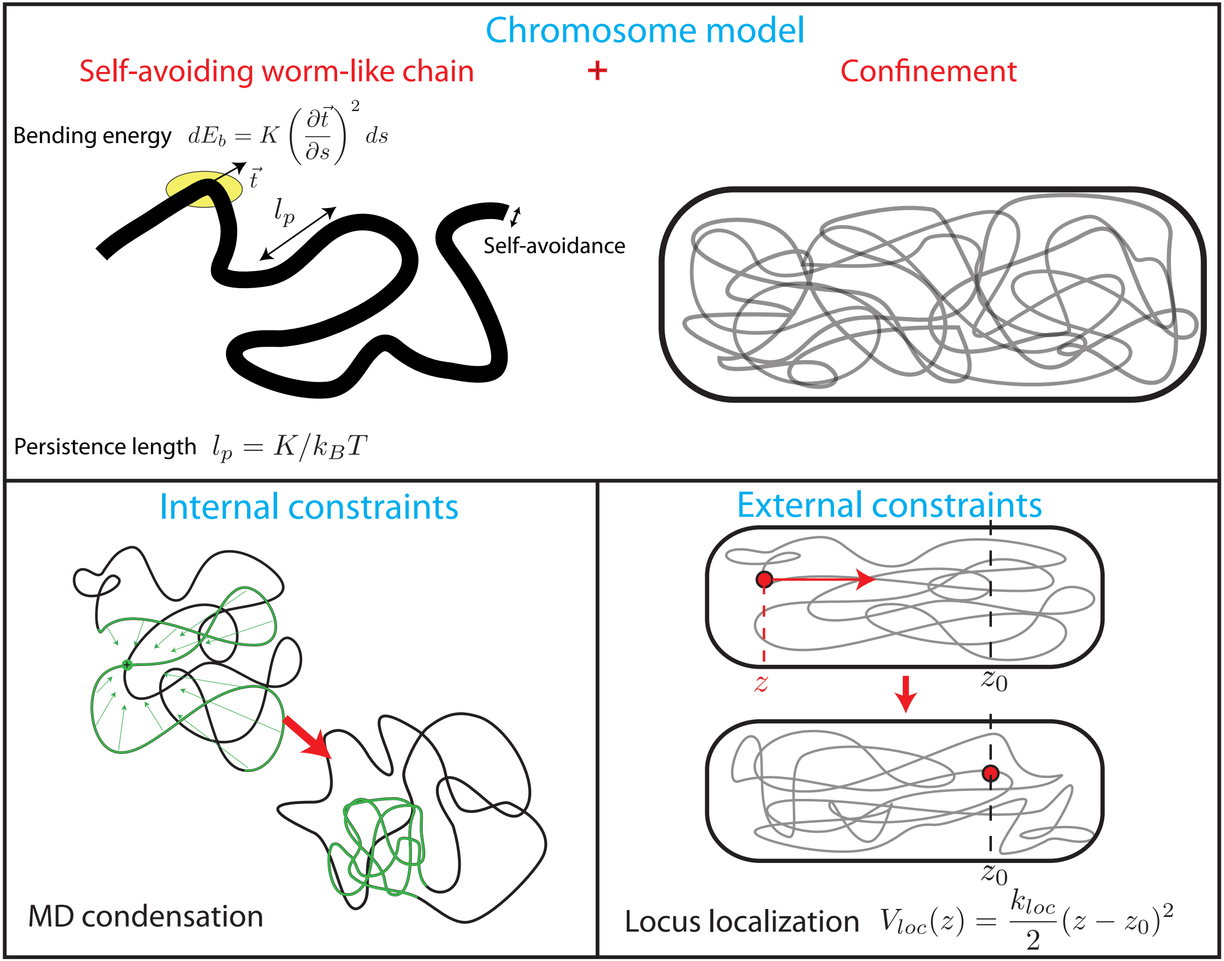

Supplementary Figure S1

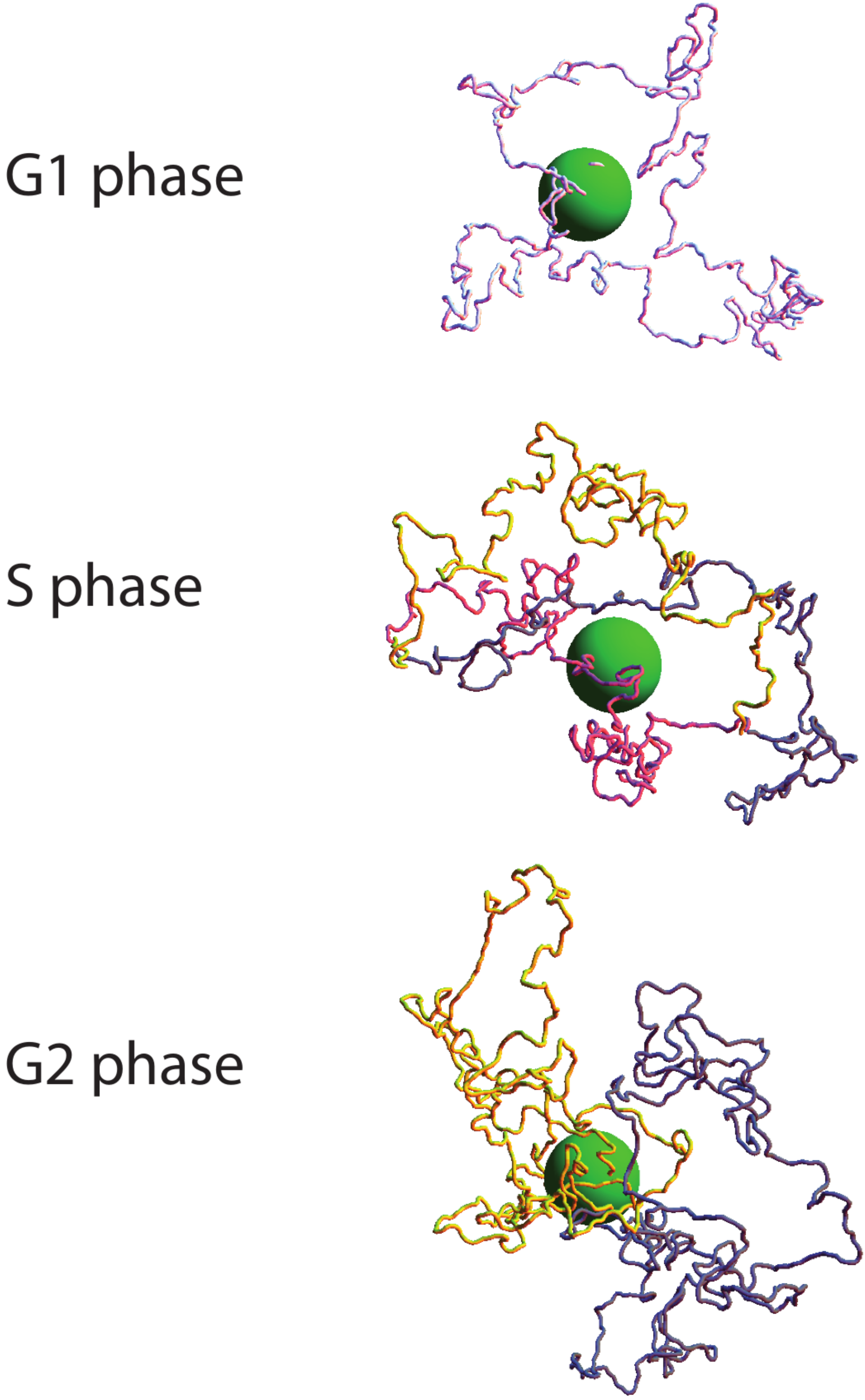

Supplementary Figure S2

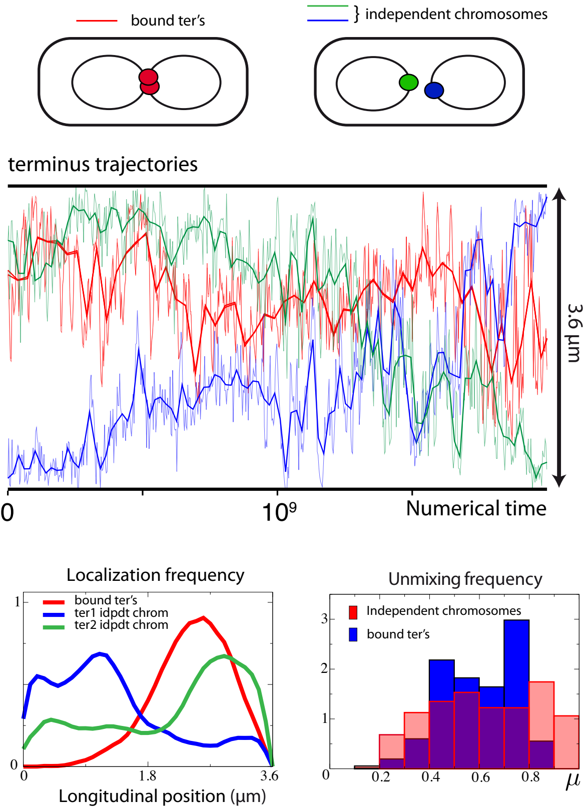

Supplementary Figure S3

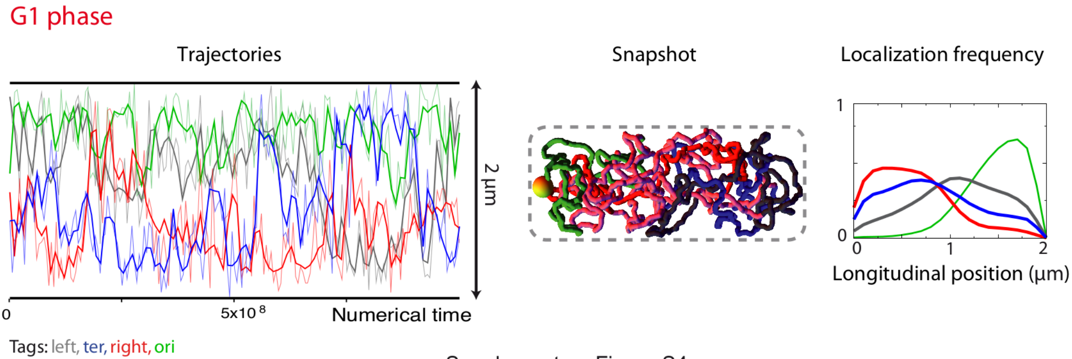

Supplementary Figure S4

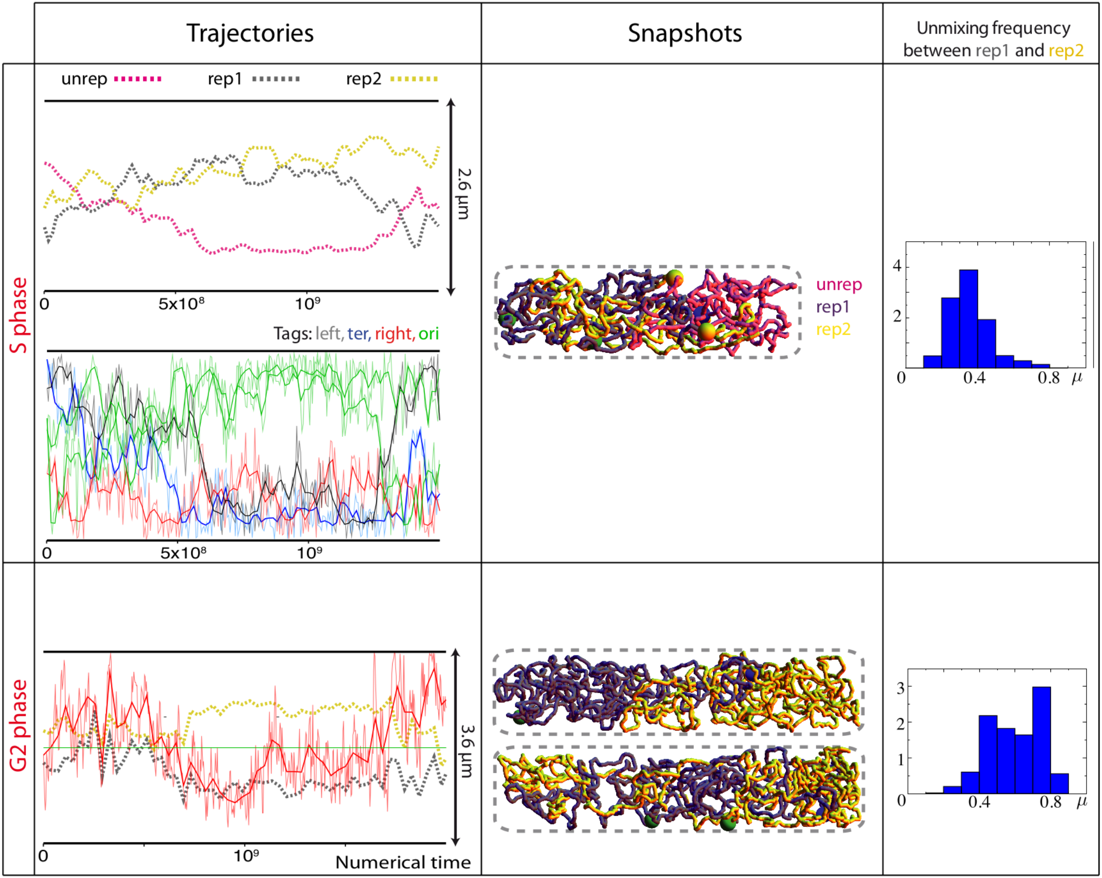

Supplementary Figure S5

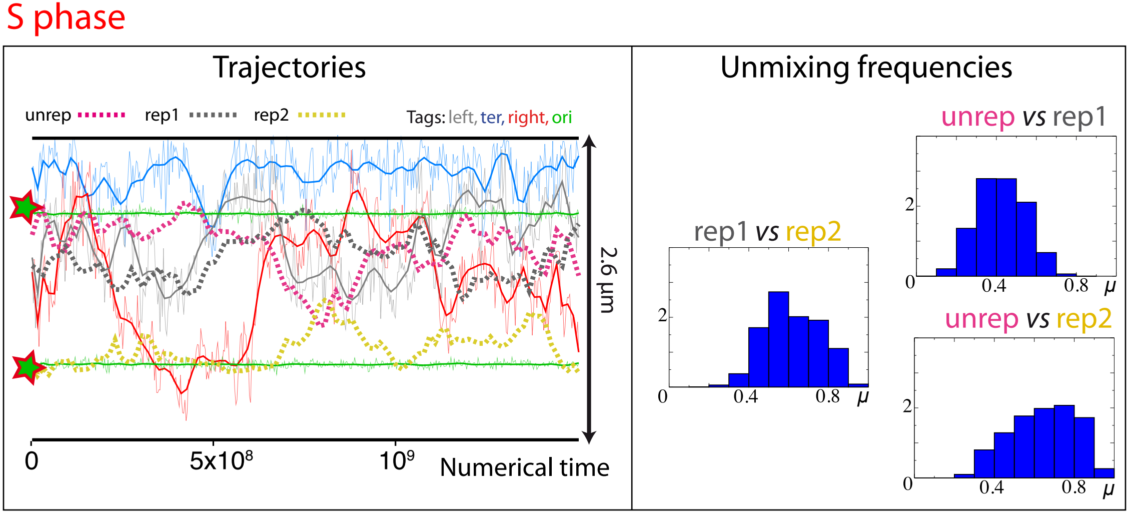

Supplementary Figure S6

Unmixing frequencies in the S phase for a homogeneous polymer in the presence of a forced localization of ori and ter

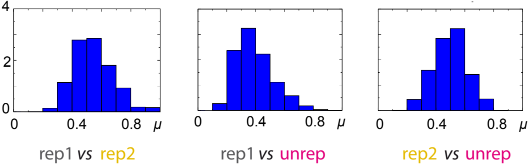

Supplementary Figure S7

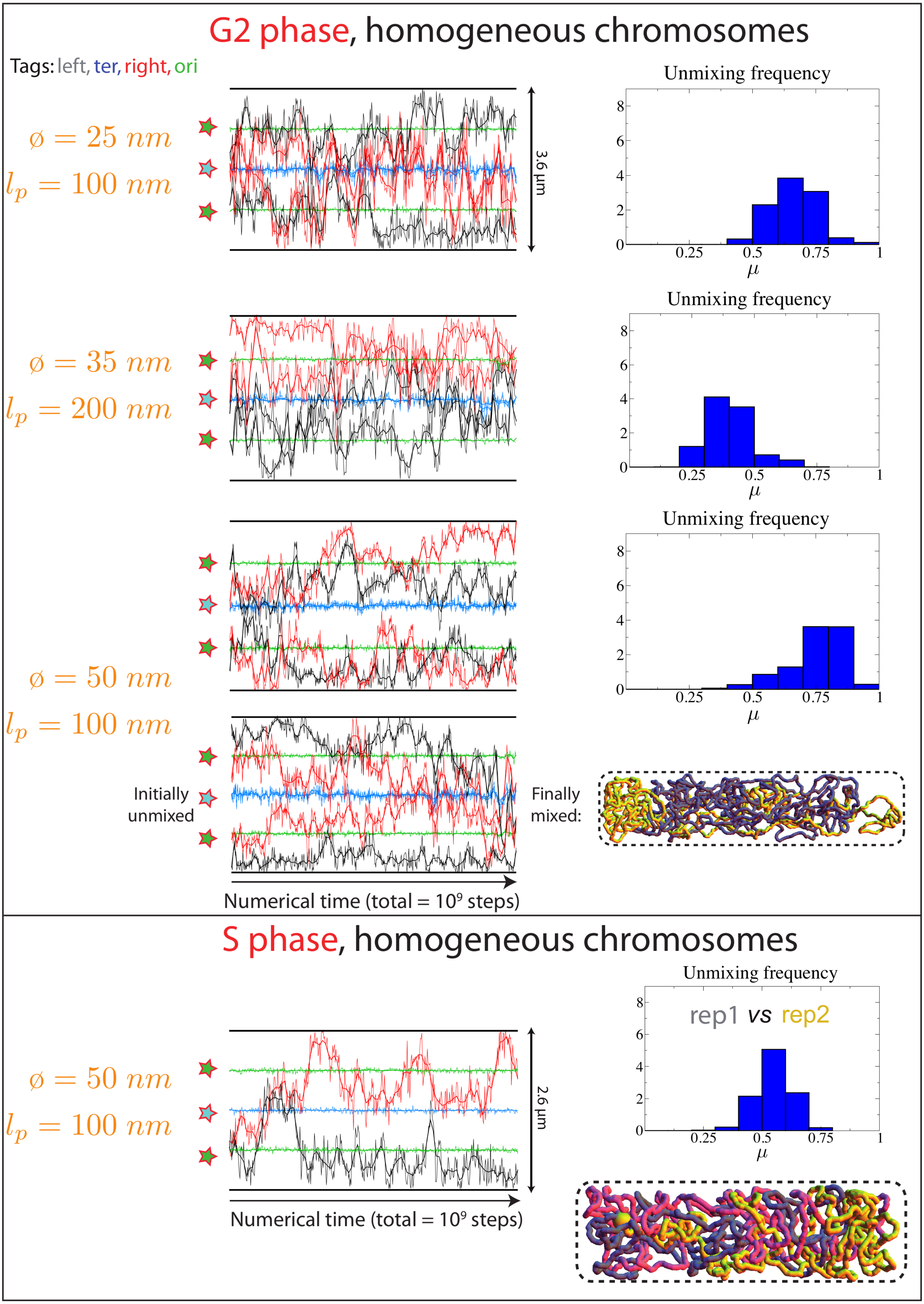

Supplementary Figure S8

G1 phase

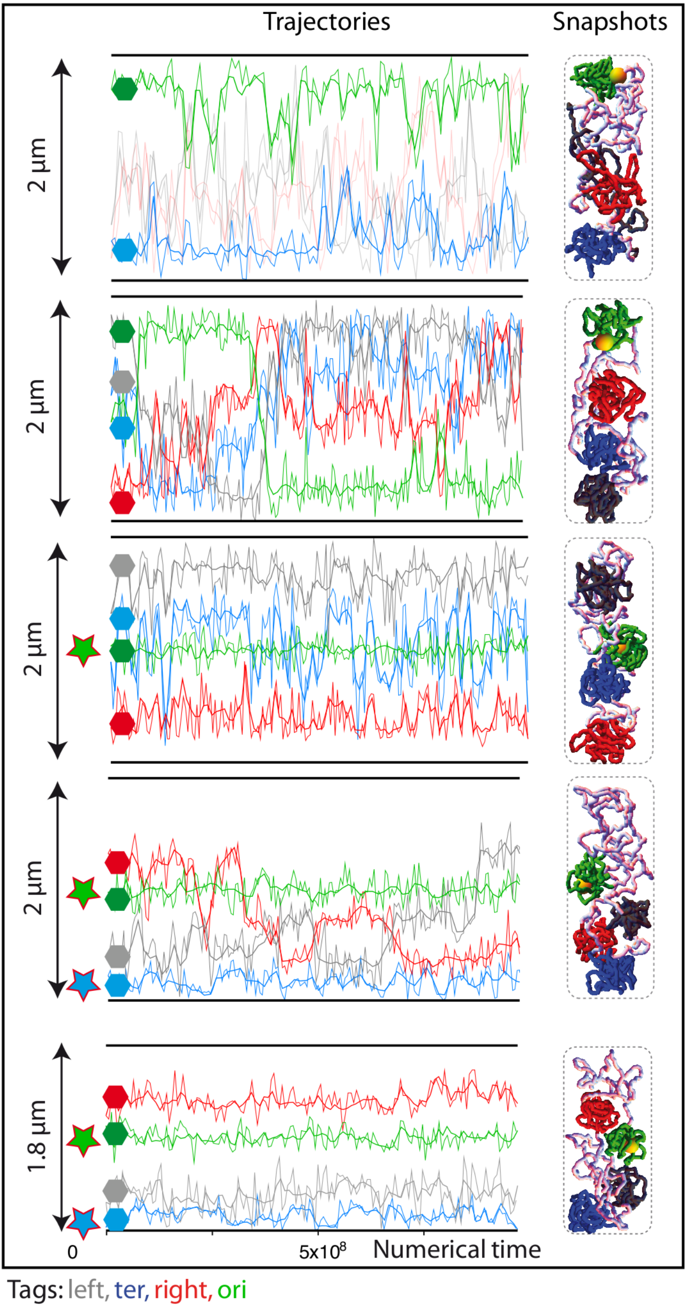

Supplementary Figure S9

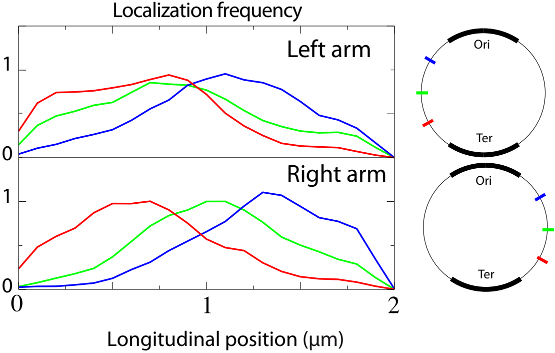

Supplementary Figure S10

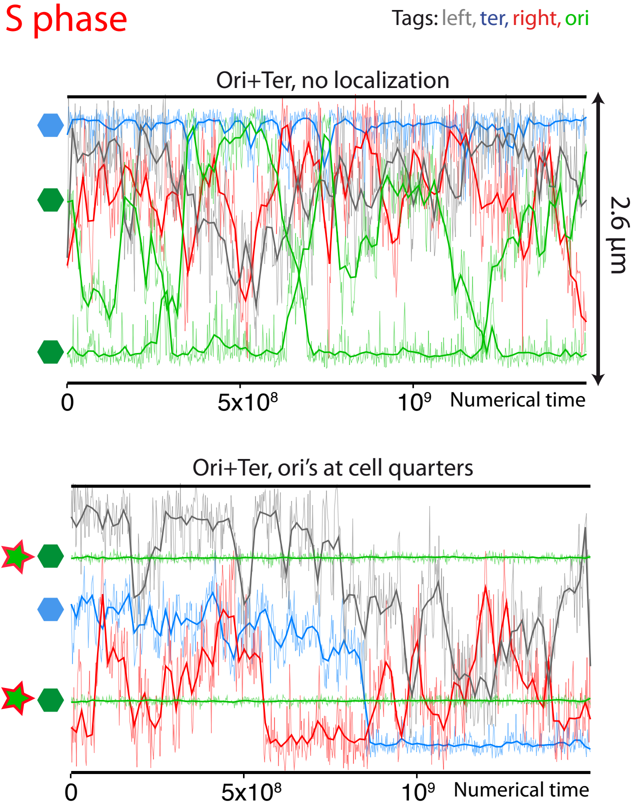

Supplementary Figure S11

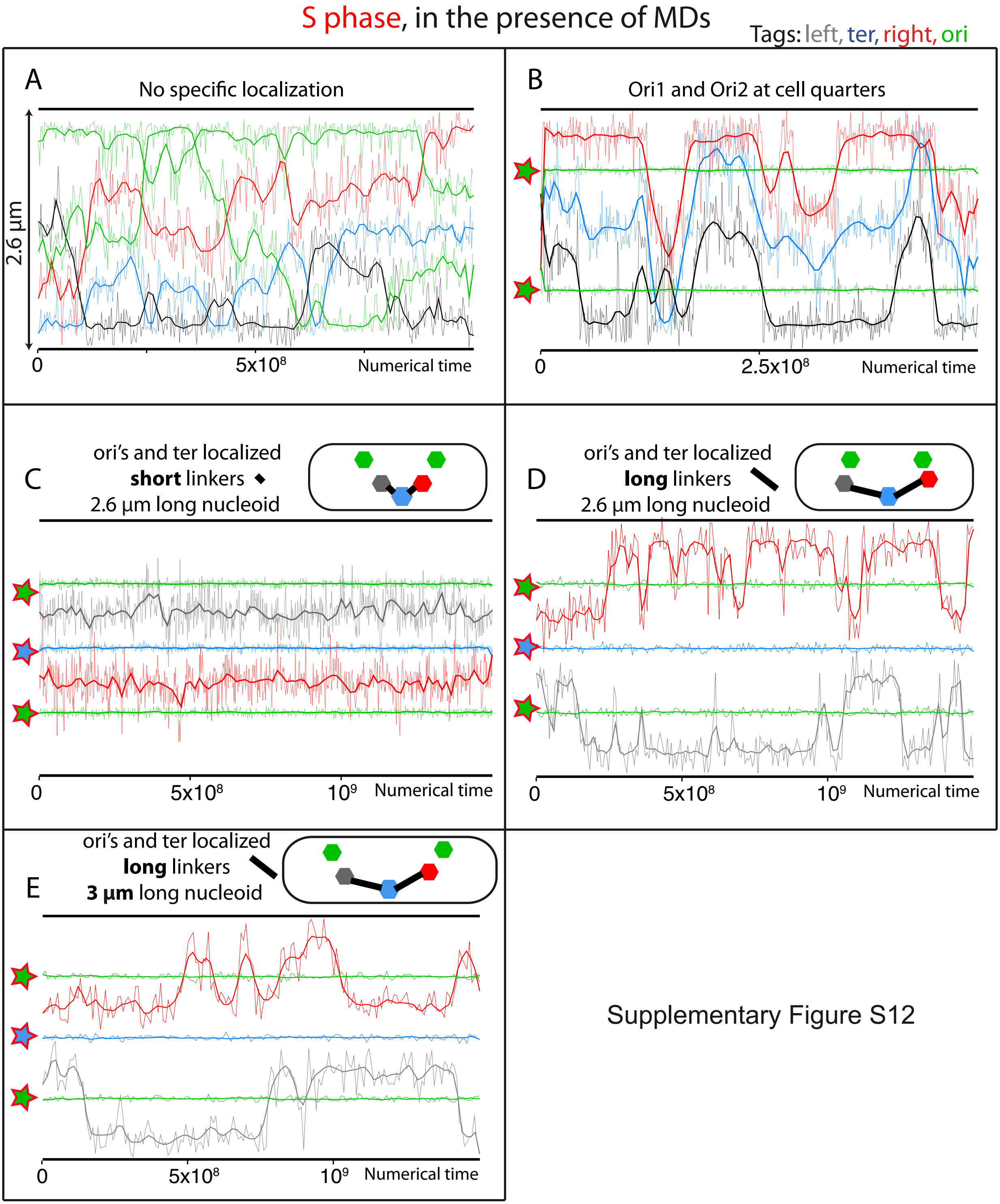

Supplementary Figure S12

G2 phase, in the presence of Ori and Ter (the right and left regions are not folded)

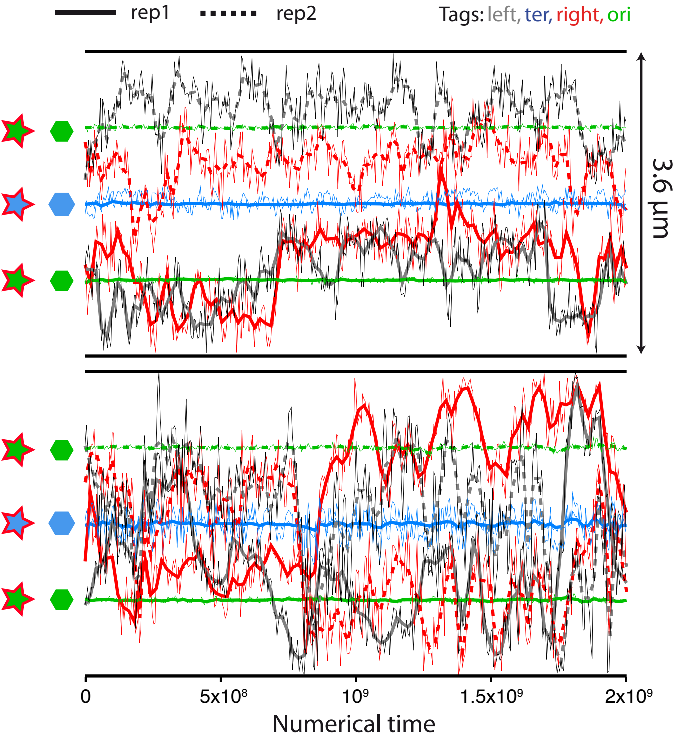

Supplementary Figure S13

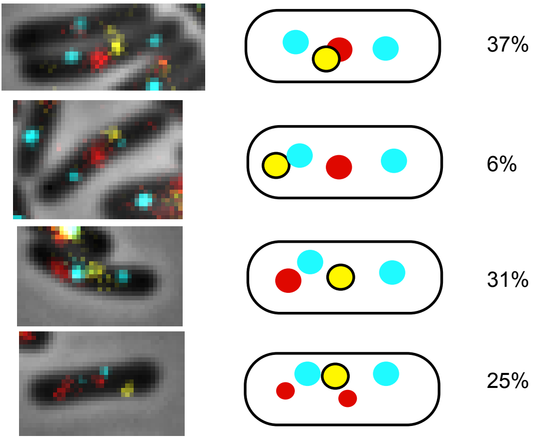

Supplementary Figure S14

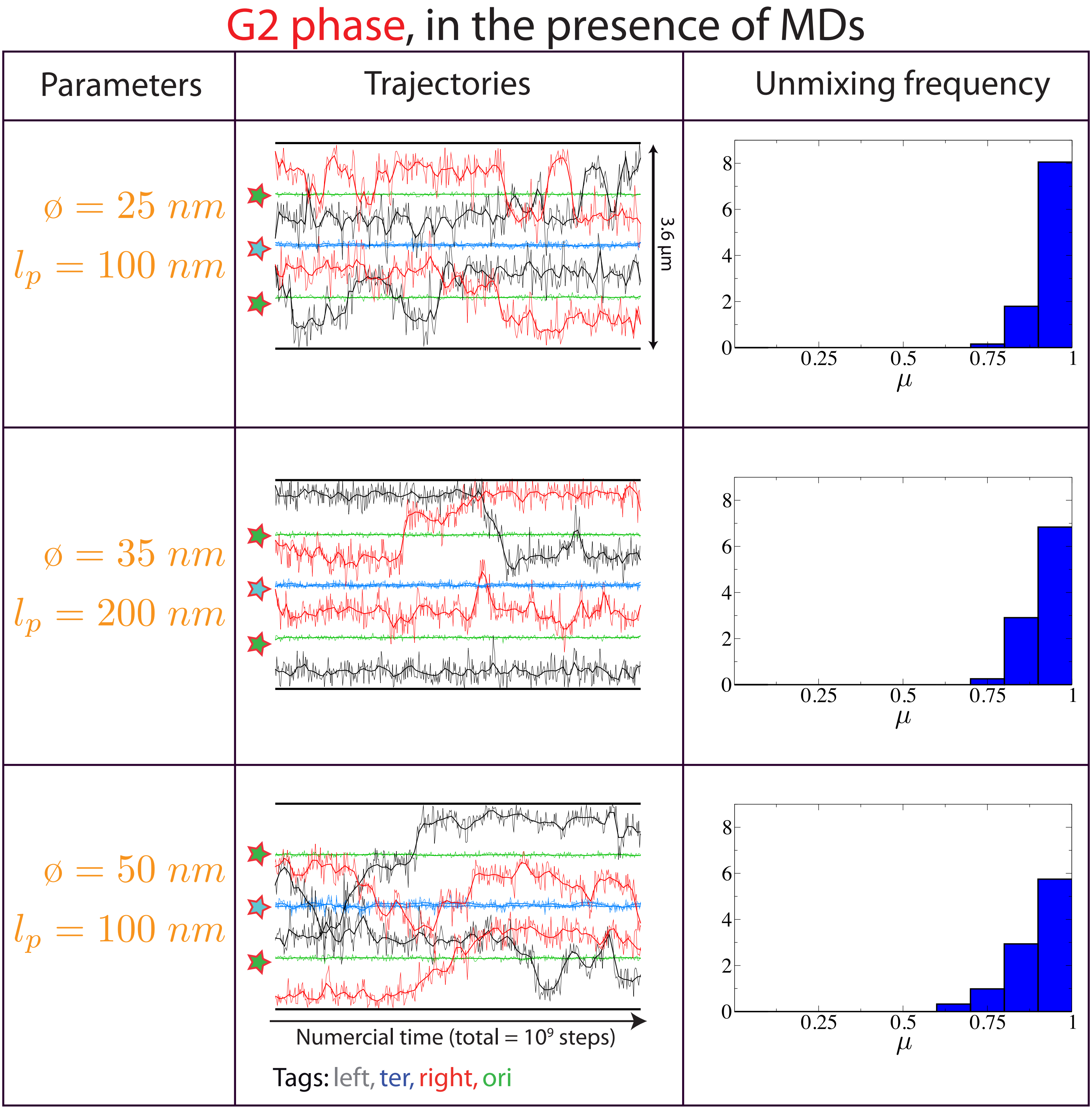

Supplementary Figure S15

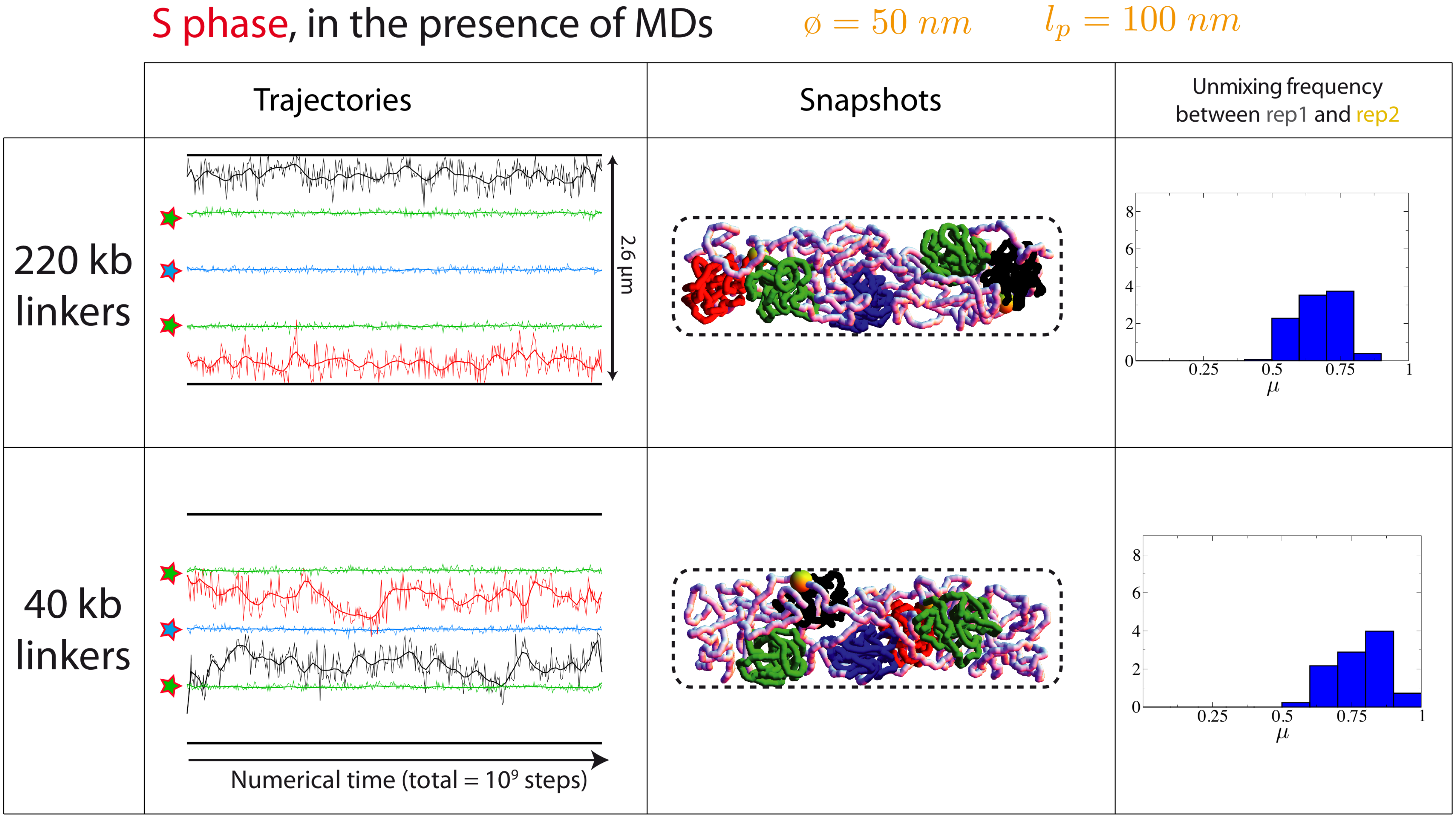

Supplementary Figure S16

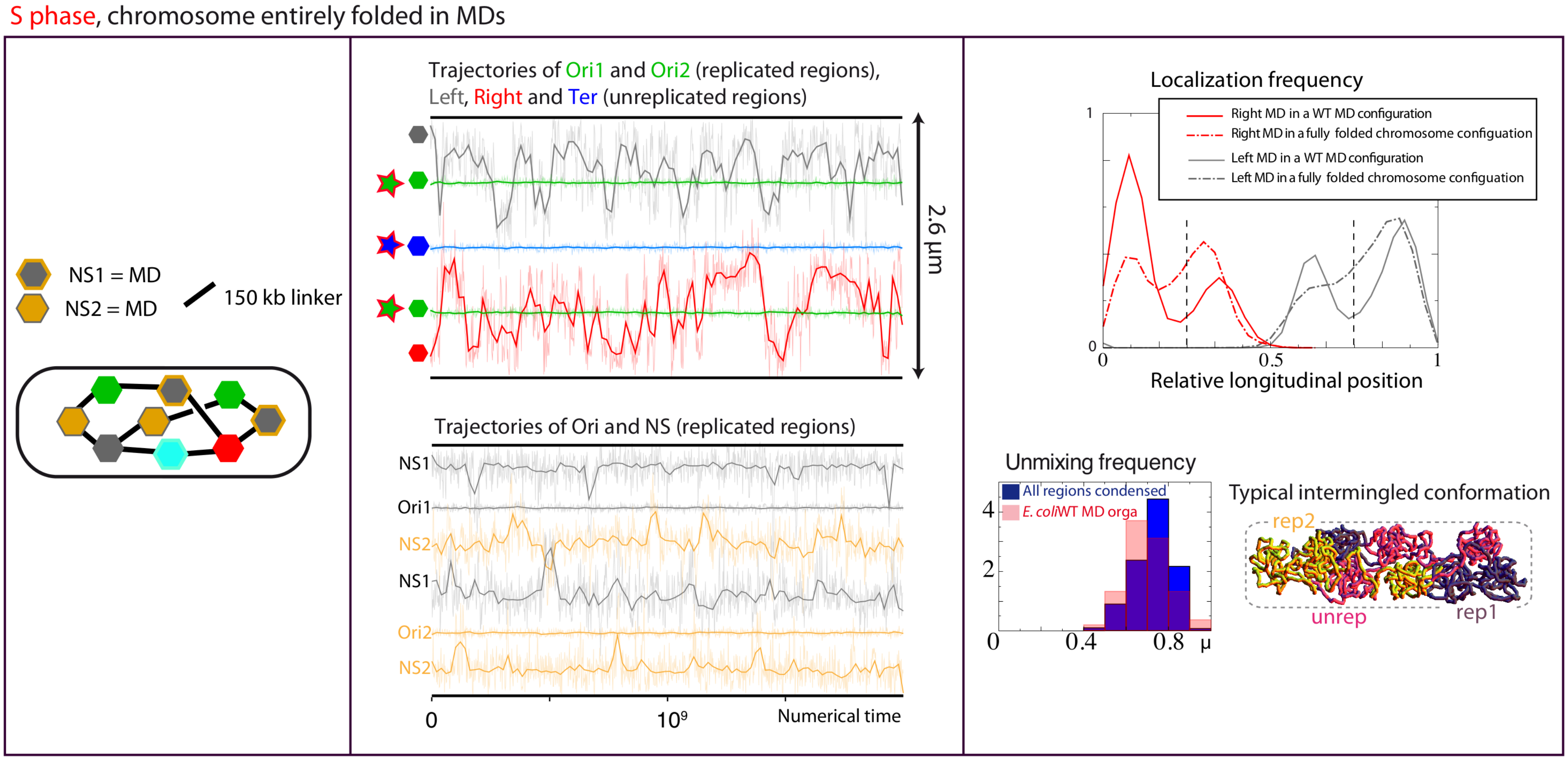

Supplementary Figure S17
